# Quality improvement interventions to prevent late-onset sepsis in premature infants: a systematic review and meta-analysis

**DOI:** 10.7717/peerj.20530

**Published:** 2026-01-02

**Authors:** Xiangtong Zhang, Zhi Wan, Kangyan Yuan, Genfeng Wu, Zhangbin Yu

**Affiliations:** 1 Department of Pediatrics, Longgang District Maternity & Child Healthcare Hospital of Shenzhen City (Longgang Maternity and Child Institute of Shantou University Medical College), Shenzhen, Guangdong, China; 2 Department of Child Health Care, Longgang District Maternity & Child Healthcare Hospital of Shenzhen City (Longgang Maternity and Child Institute of Shantou University Medical College), Shenzhen, Guangdong, China; 3 Department of Neonatology, Shenzhen People’s Hospital (The Second Clinical Medical College, Jinan University; The First Affiliated Hospital, Southern University of Science and Technology), Shenzhen, Guangdong, China

**Keywords:** Preterm infants, Quality improvement interventions, Late-onset sepsis, Systematic review, Meta-analysis

## Abstract

**Background:**

Late-onset sepsis (LOS) is a life-threatening complication in preterm infants, with reported incidence rates of 1%–30% that vary by clinical and geographical factors. Quality improvement (QI) bundles integrating infection control, nutrition, and device management show promise in reducing LOS, but evidence remains fragmented due to heterogeneous definitions and mixed study populations.

**Methods:**

A systematic review and meta-analysis of QI studies was conducted across PubMed, Embase, Cochrane Library, and Web of Science (inception to March 19th, 2025). Studies were included if they reported pre-post QI outcomes for LOS in preterm infants (gestational age < 37 weeks), with effect sizes synthesized as odds ratios (ORs) and 95% confidence intervals (CIs). Heterogeneity was evaluated using *I*^2^, with random-effects models for *I*^2^ ≥ 50%. Subgroup analyses explored LOS definition impacts (time windows: ≥48 h, ≥72 h, other) and meta-regression tested covariates (study year, sample size, quality).

**Results:**

Of 9,705 identified studies, 29 (21 for meta-analysis, *n* = 29,120 infants) met criteria. QI bundles significantly reduced LOS in very low birth weight (VLBW) infants (pooled OR = 0.47, 95% CI [0.38–0.58], *I*^2^ = 88.7%) and extremely low birth weight (ELBW) infants (OR = 0.49, 95% CI [0.29–0.83], *I*^2^ = 80.6%). Core components included multidisciplinary teams (25/29), hand hygiene (21/29), and central line management (22/29). Subgroup analysis showed varying effects by LOS definition: OR = 0.35 (95% CI [0.19–0.64]) for ≥ 48 h, OR = 0.50 (95% CI [0.39–0.64]) for ≥ 72 h, and OR = 0.71 (95% CI [0.61–0.82]) for ill-defined thresholds. Meta-regression identified no significant modifiers (all *p* > 0.0), but publication bias was detected in VLBW analyses (Egger’s test, *p* < 0.0).

**Conclusion:**

QI bundles significantly reduce LOS in preterm infants, including VLBW and ELBW subgroups, through core components like multidisciplinary teams, hand hygiene, and central line management. Given outcome variations by diagnostic criteria, standardizing LOS definitions is crucial. These bundles should be integrated into routine care globally. Future efforts should prioritize standardized reporting, antibiotic stewardship, and equitable implementation, especially in low-resource settings, by building on existing evidence and broader healthcare principles.

## Introduction

Late-onset sepsis (LOS), which is defined as the isolation of pathogenic bacteria or fungi from blood and/or cerebrospinal fluid occurring more than three days after birth, remains a critical threat to premature infants worldwide (Flannery et al., 2022). This life-threatening infection disproportionately affects preterm neonates, with studies showing it significantly elevates risks of mortality, acute brain injury, and long-term neurodevelopmental impairments such as cerebral palsy and cognitive delays (Flannery et al., 2022; Kurul et al., 2023). The clinical burden of LOS is particularly pronounced in neonatal intensive care units, where it accounts for a substantial proportion of morbidity and resource utilization.

Preterm infants are more vulnerable to LOS due to a confluence of biological and iatrogenic factors. Immature immune defenses—including deficient neutrophil function, reduced complement activity, and limited antibody production—compromise their ability to combat infections (Almeida et al., 2017). Concurrently, compromised skin barrier integrity from preterm birth, repeated invasive procedures (*e.g.*, central line insertions, blood sampling), and frequent handling in intensive care settings create portals of entry for pathogens (Collins, Weitkamp & Wynn, 2018). These factors collectively establish a high-risk milieu that exacerbates susceptibility to nosocomial and community-acquired infections.

Epidemiological data show substantial variability in LOS incidence (1%–30%) among preterm infants, with the highest rates (up to 30%) observed in very low birth weight infants (*e.g.*, Israel, Spain), while moderate rates (1%–10%) are more common in preterm infants with a wider range of gestational ages and birth weights (not limited to very low birth weight) (Wójkowska-Mach et al., 2019). This variability is influenced by clinical factors, such as premature rupture of membranes, placental abruption, maternal hypertensive disorders, intrauterine growth restriction, and stark geographical disparities (Fleischmann-Struzek et al., 2018; Letouzey et al., 2021). Alarmingly, neonates in middle-income countries face sepsis rates approximately 40 times higher than those in high-income settings, a gap attributed to resource limitations, delayed access to care, and suboptimal infection prevention protocols (Fleischmann-Struzek et al., 2018). Such disparities underscore the urgent need for scalable, evidence-based preventive strategies.

Given the significant morbidity and mortality associated with LOS, prevention has emerged as a clinical priority surpassing treatment alone. Quality improvement (QI) methodologies offer a structured framework to bridge the gap between evidence-based best practices and real-world clinical outcomes. Rooted in systematic analysis, iterative process optimization, and data-driven decision-making, QI initiatives emphasize measurable benchmarks, multidisciplinary collaboration, and continuous feedback loops to enhance healthcare delivery (Backhouse & Ogunlayi, 2020). In neonatal care, QI has demonstrated tangible benefits: successful initiatives have reduced hypothermia at birth, improved safe sleep practices to mitigate sudden unexpected neonatal death, and lowered perinatal mortality rates (Zhong et al., 2024; Kamala et al., 2025; Salm Ward & Yasin, 2022). These achievements illustrate QI’s potential to address complex, multifactorial challenges in vulnerable populations.

QI bundles integrating multiple preventive measures—including stringent infection control protocols (*e.g.*, hand hygiene, universal precautions), early enteral feeding with human milk, timely removal of central lines, standardized catheter care, and antibiotic stewardship—show promise in reducing LOS risk (Pammi & Weisman, 2015). However, clinical implementation varies widely, and consensus remains elusive regarding the optimal composition of these bundles. Current systematic reviews either focus narrowly on catheter-related bloodstream infections or include mixed populations of term and preterm infants, leaving a critical knowledge gap about QI strategies tailored to preterm neonates (Huskins, 2012; Payne et al., 2018). Moreover, a lack of quantitative synthesis across studies hinders the development of evidence-based guidelines. This systematic review and meta-analysis aim to synthesize available evidence on the efficacy and safety of QI bundles in reducing LOS among premature infants, identify key components associated with successful outcomes, and inform clinical practice and resource allocation to mitigate the global burden of this devastating complication.

## Materials and Methods

This systematic review and meta-analysis adhered to the Preferred Reporting Items for Systematic Reviews and Meta-Analyses (PRISMA) guidelines. The study protocol was prospectively registered in the PROSPERO database (Registration ID: CRD420251014631) and did not require ethical approval, as it involved a secondary analysis of publicly available data.

### Data sources and search strategy

A comprehensive search was conducted across four electronic databases—PubMed, Embase, Cochrane Library, and Web of Science—from their inception to March 19th, 2025. The initial searches were unrestricted to maximize sensitivity, and detailed search strategies for each database are provided in Appendix S1.

### Inclusion and exclusion criteria

Studies were selected based on the PICOS framework: (1) Population: Preterm infants with a gestational age (GA) of less than 37 weeks, including very low birth weight (VLBW) and extremely low birth weight (ELBW) infants. (2) Intervention: Implementation of QI bundles aimed at preventing LOS. (3) Comparison: Pre-QI cohorts from the same institution(s). (4) Outcome: Incidence of LOS during neonatal intensive care unit (NICU) hospitalization, defined as sepsis occurring more than 72 h after birth. (5) Study design: Quality improvement projects that report pre- and post-intervention LOS rates. Exclusion criteria included: (1) Studies involving term infants (GA of 37 weeks or greater). (2) Non-QI designs (*e.g.*, randomized trials of single interventions). (3) Inability to extract LOS incidence data. (4) Publications limited to conference abstracts.

### Study selection and data extraction

Two independent reviewers (XZ and ZW) screened the titles and abstracts and then assessed the full texts of potentially eligible studies from March 19th to April 10th, 2025. Discrepancies were resolved through discussion or consultation with a third reviewer (GW). Data extraction was conducted from April 11th to April 20th, 2025 using a standardized form to capture study characteristics, including the author, year, country, duration, participant demographics, definitions of LOS, pre- and post-intervention sample sizes, LOS events, and components of the QI bundle. Disagreements were resolved through discussion with a third reviewer (ZY).

### Quality evaluation and risk bias assessment

The methodological quality was assessed by two investigators (XZ and KY) using the Quality Improvement Minimum Quality Criteria Set (QI-MQCS) (Hempel et al., 2015), which evaluates 16 domains, including intervention description and implementation strategies. Each domain was assigned a score of 0 (unmet) or 1 (met), with total scores classified as high (>10), moderate (7–10), or low quality (<7). Any disagreements were resolved through consensus.

### Statistical analysis

When synthesizing effect sizes, preterm infant data were categorized into three subgroups based on both birth weight (BW) and GA: ELBW (BW < 1,000 g or GA < 29 weeks), VLBW (BW < 1,500 g or GA < 33 weeks), and LBW (low birth weight; BW < 2,500 g or GA < 37 weeks). Only studies providing binary variable data (or derived binary variables) for late-onset sepsis were included; multi-group studies contributed data to respective subgroups. Outcomes were summarized as odds ratios (ORs) with 95% CIs (forest plots), analyzed *via* Stata 17.0. Heterogeneity was quantified by *I*^2^: random-effects models for *I*^2^ ≥ 50%, fixed-effects for *I*^2^ < 50%. Sensitivity analyses explored heterogeneity sources, while meta-regression was performed for subgroups with ≥10 studies (minimal sample size for stable estimates). Categorical covariates were dummy-coded: study year (pre-2015 (reference group) *vs.* post-2015), population size (<500 (reference group) *vs.* ≥500), clinical setting (single-center (reference group) *vs.* multicenter), and study quality (<13 (reference group, Newcastle-Ottawa Scale) *vs.* ≥13). The weighted least squares model used log-transformed OR (lnOR) as the dependent variable, weighted by the inverse variance of lnOR. Coefficients (β) represented lnOR differences *vs.* reference groups, with e^β^ interpreting OR ratios. Subgroup analyses of LOS time windows (≥48 h, ≥72 h, other definitions like ≥10 days or unspecified) were conducted separately. Due to non-standardized “other” definitions and sample dispersion risks, these groups were not included in meta-regression, allowing direct effect size comparisons under distinct diagnostic criteria while avoiding model bias. Publication bias was assessed *via* funnel plots for subgroups with ≥10 studies (*p* < 0.05 for significance).

## Results

### Study selection

**Figure 1:**
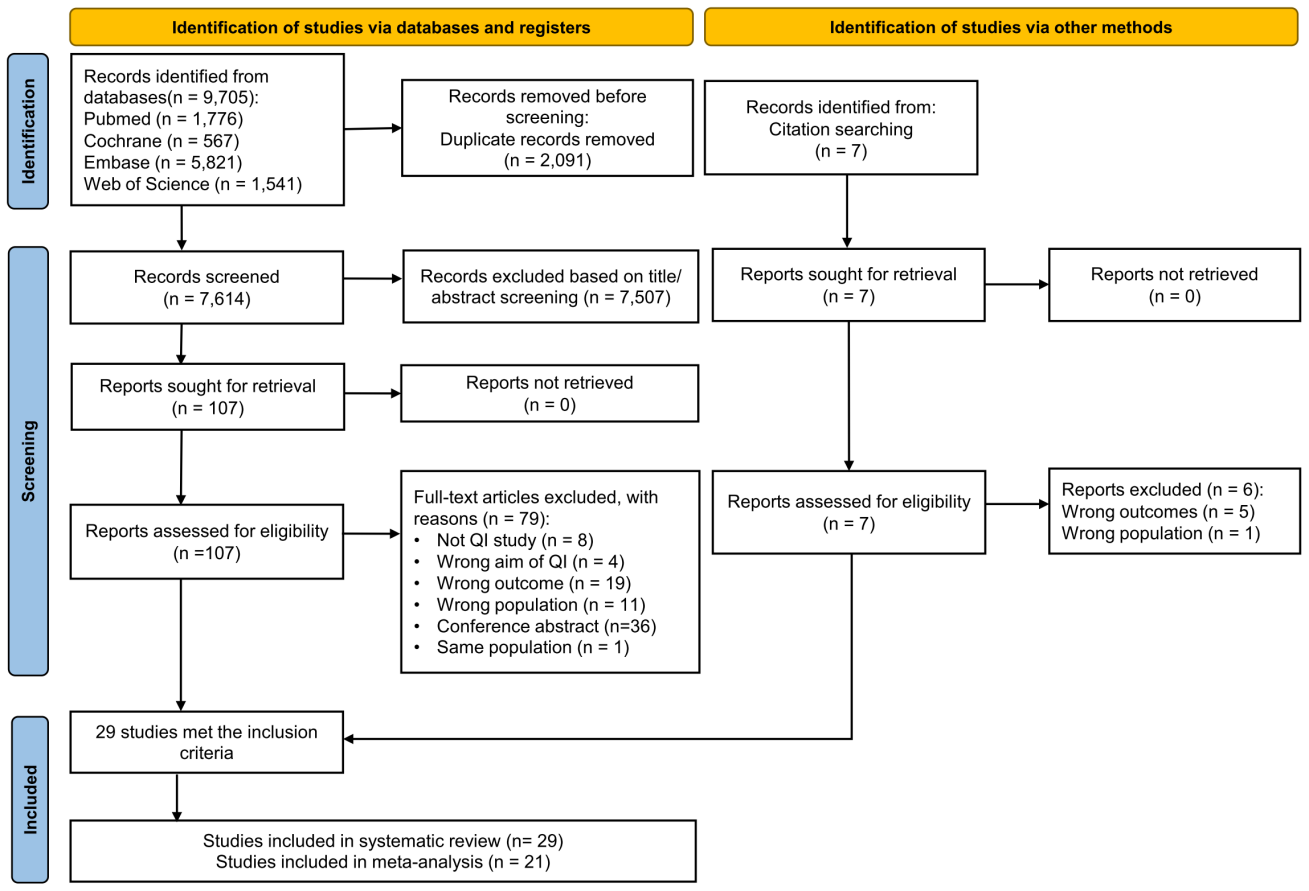
PRISMA flowchart summarizing the article selection process.

A total of 9,705 articles were retrieved from the electronic databases, including PubMed (1,776), Embase (5,821), the Cochrane Library (566), and Web of Science (1,541). Of the 7,614 articles remaining after removing duplicates, the majority were excluded after the first screening based on titles or abstracts. The full texts of 107 articles and seven additionally identified studies were scanned. Among these, 85 articles were excluded (Table S1) due to reasons such as not being quality improvement studies (eight), including newborns with a gestational age greater than 32 weeks (twelve), aim not related to quality improvement (four), no reported outcome on late-onset sepsis rate (24), only conference abstracts available (36), and being from the same population (one). Following the full-text screening, 29 articles met the criteria for qualitative synthesis. Among these, 21 articles were deemed suitable for quantitative synthesis through meta-analysis. A detailed flowchart illustrating the screening and selection process is provided in Fig. 1, following PRISMA guidelines.

### Study characteristics

The included 29 studies originated from diverse locations, with eleven from the USA (Aly et al., 2005; Bizzarro et al., 2010; Bloom et al., 2003; Horbar et al., 2001; Kaplan et al., 2011; Kilbride et al., 2003; Morris, Cleary & Soliman, 2015; Mwananyanda et al., 2019; Payne et al., 2012; Payne et al., 2010; Wicker et al., 2011; Wirtschafter et al., 2011), three from Australia (Andersen et al., 2005; Bowen et al., 2017; Gill et al., 2011), three from Canada (Lee et al., 2015; Lee et al., 2009; Shah et al., 2019), three from India (Balla et al., 2018; Batthula, Somnath & Datta, 2021; Rakshit et al., 2023), two from the UK (Davis et al., 2016; Sinha et al., 2016) and one each from Bangladesh (Darmstadt et al., 2005), China (Bi et al., 2022), Germany (Salm et al., 2016), Israel (Peleg et al., 2019), Korea (Shin et al., 2024), Portugal (Almeida et al., 2017) and Zambia (Mwananyanda et al., 2019). The publication timeline of the selected studies spans from 1994 to 2023. Nearly half of the quality improvement initiatives (15 out of 29) were single-center studies, while the other 14 were conducted as multicenter projects. The sample sizes varied considerably, ranging from 68 to 12,245 (total = 38,902 infants). Table 1 displays the characteristics of the included studies.

**Table 1:** Characteristics of included studies.

**References**	**Location**	**Setting**	**Study period**	**Size**	**GA/BW**	**Definition of LOS**	**Results (rate of LOS, %; pre- *vs.* post- QI)**
Horbar et al. (2001)	USA	multi	1994–1997	1,517	BW <1,500 g	The occurrence of one or more infections after the third day of life with either CONS or another bacterial pathogen	26.3 to 20.9 (decrease)
Bloom et al. (2003)	USA	multi	1997–1999	68	GA < 28 w or BW < 1,500 g	Sepsis occurring 3 or more days after birth	3.8 to 2.9 episodes per 1,000 patient days (decrease)
Kilbride et al. (2003)	USA	multi	1997–2000	NA	BW < 1,500 g	A positive culture from blood or CSF, signs of generalized infection, and treatment with 5 days or more of antibiotics	24.6 to 16.4 (decrease)
Aly et al. (2005)	USA	single	1998–2003	536	BW < 2,500 g	A culture-proven infection episode in the bloodstream, excluding contaminants if antibiotic treatment is discontinued within 72 h	25.5 to 2.20 (decrease)
Andersen et al. (2005)	Australia	single	2002–2003	174	BW < 1,500 g	Positive blood cultures occurring after 48 h of birth	21.0 to 9.0 (decrease)
Darmstadt et al. (2005)	Bangladesh	single	1998–2003	NA	GA < 33 w	A positive blood or CSF culture obtained more than 3 days after hospitalization	declines in cases of culture-proven (61%) sepsis
Lee et al. (2009)	Canada	multi	2002–2005	1,005	GA < 33 w	The growth of 1 or more organisms, including CONS, in blood or CSF cultures taken after 48 h of an infant’s admission with clinical suspicion of infection	25.4 to 17.4 (decrease)
Bizzarro et al. (2010)	USA	single	2005–2007	576	mean GA < 36 w, mean BW < 2,500 g	BSI with onset at more than 72 h of life	5.84 to 1.42 cases per 1,000 patient-days (decrease)
Payne et al. (2010)	USA	multi	1998–2006	2,705	BW < 1,500 g	The isolation of CONS or other bacterial pathogen from the blood or CSF after postnatal day 3	18 to 15 (decrease)
Gill et al. (2011)	Australia	multi	2003–2008	990	GA < 29 w	A positive culture of a single organism in blood or CSF, combined with a CRP level > 20 mg/L within 72 h of the positive culture.	13 to 7 cases per 1,000 patient-days (decrease)
Kaplan et al. (2011)	USA	multi	2008–2009	NA.	GA between 22 and 29 w	Either a positive bacterial culture of blood or CSF obtained 72 h or more after birth, or a positive blood or CSF culture for CONS obtained 72 h or more after birth, associated with generalized symptoms of illness and with the infant receiving antibiotics for 5 days or more	18.2 to 14.0 (decrease)
Wicker et al. (2011)	USA	multi	2001–2008	637	BW < 1,500 g	A positive blood and/or CSF culture after 3 days of life	37.7 to 22.7 (decrease)
Wirtschafter et al. (2011)	USA	single	2002–2006	12,245	BW < 1,500 g	A late bacterial or CONS infection diagnosed after the age of 3 days by positive blood/CSF culture(s) and clinical criteria, with additional criteria applied when CONS is recovered from a culture	18.2 to 15.8 (decrease)
Payne et al. (2012)	USA	single	2000–2009	3,470	BW < 1,500 g	Bloodstream or meningeal infections occurring after postnatal day 3 (*i.e.*, 72 h after birth)	27.4 to 16.3 (decrease)
Lee et al. (2015)	Canada	single	2005–2007	345	GA < 33 w	The growth of one or more organisms in one blood or CSF culture obtained after 48 h of admission in a symptomatic infant	20.8 to 13.1 (decrease)
Morris, Cleary & Soliman (2015)	USA	single	2008–2013	222	GA < 29 w	Infections occurring after birth, likely referring to sepsis developing beyond the early neonatal period	39.3 to 19.4 (decrease)
Davis et al. (2016)	UK	single	2002–2011	757	VLBW	Infection commencing after 48–72 h of age	38.3 to 18.8 (decrease)
Salm et al. (2016)	Germany	multi	2009–2011	4,556	BW < 1,500 g	Clinical BSI without pathogen detection (where the attending physician begins appropriate antimicrobial therapy for sepsis for at least 5 days, no pathogen is detected in the blood culture or no blood culture is performed, and no other infection can explain the symptoms), microbiologically confirmed BSIs with pathogen detection in the blood culture other than CONS, and microbiologically confirmed BSI with CONS	24.9 to 19.3 (decrease)
Sinha et al. (2016)	UK	single	2007–2012	309	GA < 32 w	BSI acquired 48 h after birth	60.5 to 12.7 (decrease)
Almeida et al. (2017)	Portugal	single	2007–2013	348	BW < 1,500 g	Sepsis occurring at least 72 h after birth in neonates	43.0 to 21.6 (decrease)
Bowen et al. (2017)	Australia	multi	2012–2014	719	GA < 29 w	A definite pathogen is found in blood culture. Or, if a possible contaminant (like CONS) grows in the blood, and the neonate receives antibiotic treatment for 96 h or more (or dies within 96 h), plus the same organism grows on a repeat culture, or there are one or more abnormal laboratory markers or clinical features indicating systemic infection.	24.9 to 12.3 (decrease)
Balla et al. (2018)	India	single	2015–2016	1,565	mean GA 33–35 weeks, mean BW 2,000–2,100 g	A laboratory-confirmed BSI not secondary to infection at another site, excluding positive blood cultures on admission and contaminated samples	7.3 to 2.3 per 1,000 patient-days (decrease)
Mwananyanda et al. (2019)	Zambia	multi	2015-2017	1257	BW < 2,500 g	Hospital-associated sepsis with an onset ≥ 3 days after NICU admission	31.6 to 10.6 (decrease)
Peleg et al. (2019)	Israel	single	2015	385	GA < 33 w	Proven sepsis that occurs after the first few days of life	17.7 to 10.4 (decrease)
Shah et al. (2019)	Canada	multi	2013–2017	3024	GA < 29 w	A positive blood culture or CSF culture in a symptomatic infant after 2 days of age	24 to 21 (decrease)
Batthula, Somnath & Datta (2021)	India	single	2018–2019	84	BW < 1,500 g	The onset of sepsis’s signs and symptoms after 72 h of birth	36.4 to 23.5 (decrease)
Bi et al. (2022)	China	multi	2018–2020	750	BW < 1,500 g	A condition diagnosed by the clinical manifestations of systemic infection after 3 days of birth and abnormal values for 2 or more of the following non-specific infection indicators: WBC<5 ×109/L or WBC >20 ×109/L, CRP ≥ 10 mg/L, PLTs≤100 ×109/L, and PCT > 2 ng/ml	33.0 to 21.9 (decrease)
Rakshit et al. (2023)	India	multi	2021–2022	463	GA < 32 w	BSI with a date of event on day 3 or later	74.78 to 37.49 (decrease)
Shin et al. (2024)	Korea	single	2020–2023	195	BW < 1,500 g	Sepsis that occurs after 10 days of life, at least 3 days after PICC insertion	25.6 to 10.5 (decrease)

**Notes.**

BSI bloodstream infection BW birth weight CONS coagulase - negative staphylococcus CRP C-reactive protein CSF cerebrospinal fluid GA gestational age PICC peripherally inserted central catheter PLT platelet PCT procalcitonin

### Quality and risk of bias assessment

The included studies were assessed using the Quality Improvement Minimum Quality Criteria Set (QI-MQCS). The results showed study scores ranging from 10 to 16, with two categorized as medium quality and 27 as high quality, detailed in Appendix S2. Each study met the minimum quality requirements in eight of the 16 evaluated domains (organizational motivation, rationale behind the intervention, detailed intervention description, study design, data sources, intervention timing, observed health outcomes, and limitations). However, a predominant issue in most studies was the lack of details on the potential for scalability or replication (spread: 20 out of 29 studies). Similarly, descriptions lacked details on the spreading of interventions (sustainability: 15 out of 29 studies), description of the control group (comparator description: 10 out of 29 studies), and characteristics of the organization (organization characteristics: eight of 29 studies).

### Bundle components

A total of 20 components were identified across the 29 studies, with the number of components per bundle varying. The number of components ranged from three to sixteen. The most common components included a multidisciplinary team and opinion leaders (25/29), education and training of medical staff (23/29), line entry and maintenance management (22/29), audit and feedback (22/29), hand hygiene optimization (21/29), and diagnostic process (21/29). These were followed by skin preparation (15/29), environmental improvements to the NICU (14/29), chlorhexidine use (14/29), daily line need assessment (11/29), judicious use of antibiotics (11/29), checklists/toolkit (11/29), respiratory support strategies (11/29), dressing change management (11/29), closed vascular systems (10/29), scrub the hub (9/29), earlier enteral feeds (7/29), two-person technique (6/29), thermoregulation (3/29) and central line trolley/kit (2/29). Table 2 displays the 20 components included in the QI bundles.

**Table 2:** Interventions included in the QI bundles.

**References**	(1)	(2)	(3)	(4)	(5)	(6)	(7)	(8)	(9)	(10)	(11)	(12)	(13)	(14)	(15)	(16)	(17)	(18)	(19)	(20)
Horbar et al. (2001)	+	+	+	+	+	+	+						+				+			
Bloom et al. (2003)	+		+	+	+		+	+		+			+	+	+			+		
Kilbride et al. (2003)	+	+	+	+	+	+	+		+		+	+			+	+				
Aly et al. (2005)	+		+	+		+	+							+	+	+		+		
Andersen et al. (2005)	+	+	+		+	+	+		+	+	+	+		+			+			+
Darmstadt et al. (2005)	+	+	+		+	+		+	+		+								+	
Lee et al. (2009)	+	+	+	+		+							+							
Bizzarro et al. (2010)	+	+	+	+	+	+	+		+	+				+	+		+	+		
Payne et al. (2010)	+			+									+							
Gill et al. (2011)	+		+	+	+	+	+	+	+				+	+	+					
Kaplan et al. (2011)	+	+	+	+	+		+		+	+		+		+	+	+				
Wicker et al. (2011)	+	+	+		+	+	+	+	+	+	+			+		+	+	+		
Wirtschafter et al. (2011)	+	+	+		+	+						+			+		+			
Payne et al. (2012)	+	+	+	+	+		+		+				+	+	+	+	+			
Lee et al. (2015)	+	+	+	+	+		+	+	+		+		+	+			+			
Morris, Cleary & Soliman (2015)	+	+		+		+		+			+	+	+							
Davis et al. (2016)	+		+	+	+	+		+							+					
Salm et al. (2016)		+	+		+					+	+	+								
Sinha et al. (2016)		+	+	+	+	+	+	+	+		+					+				
Almeida et al. (2017)	+	+	+		+	+	+		+	+					+					
Bowen et al. (2017)	+	+	+	+	+	+	+	+	+	+	+	+		+		+		+		+
Balla et al. (2018)	+	+	+	+	+	+			+	+		+				+		+		
Mwananyanda et al. (2019)		+			+	+	+	+	+											
Peleg et al. (2019)	+	+		+		+		+			+	+	+						+	
Shah et al. (2019)	+	+		+									+							
Batthula, Somnath & Datta (2021)	+	+	+	+	+	+		+		+		+		+		+				
Bi et al. (2022)	+	+		+								+	+						+	
Rakshit et al. (2023)	+	+		+	+	+		+			+									
Shin et al. (2024)			+	+		+		+		+										

**Notes.**

(1) Multidisciplinary team and opinion leaders; (2) Education and training; (3) Line entry and maintenance management; (4) Audit and feedback; (5) Hand hygiene optimization; (6) Diagnostic process; (7) Skin preparation; (8) Environmental improvements to the NICU; (9) Chlorhexidine use; (10) Daily line need assessment; (11) Judicious use of antibiotics; (12) Checklists/toolkit; (13) Respiratory support strategies; (14) Dressing change management; (15) Closed vascular systems; (16) Scrub the hub; (17) Earlier enteral feeds; (18) Two person technique; (19) Thermoregulation; (20) Central line trolley/kit.

### Definition of LOS

Significant heterogeneity was observed in the definitions of LOS across the 29 included studies (Table 1). The majority (16/29, 55.2%) of the studies adopted a 72-hour time threshold to distinguish LOS from early-onset sepsis, while six studies (20.7%) defined LOS as infections occurring after 48 h of birth. Notably, six studies (20.7%) failed to specify the time threshold for LOS, and one study (3.4%) used a 10-day postnatal period as the cutoff.

Regarding diagnostic criteria, 11 studies (37.9%) defined LOS based on identifying pathogens in either blood or cerebrospinal fluid. In contrast, 5 studies (17.2%) relied solely on blood-based pathogen detection for diagnosis, and 13 studies (44.8%) did not provide explicit descriptions of their diagnostic criteria. This variability in both temporal thresholds and diagnostic methods highlights potential sources of heterogeneity that may impact the comparability and synthesis of the study results.

### Decrease in LOS incidence

Through a comprehensive systematic review, 29 eligible studies were initially identified. Among them, 21 studies were ultimately included in the quantitative synthesis, collectively involving 29,120 preterm infants. In these 21 included studies, the incidence rates of LOS before and after QI interventions could be successfully extracted. For each study, the incidence was calculated with the numerator being the total number of LOS events (diagnosed per the study’s specified criteria) and the denominator being the total number of preterm infants enrolled in its QI program, with consistent definitions applied to both pre-QI and post-QI periods. Conversely, eight studies were excluded from the meta-analysis. Specifically, four of these excluded studies were removed owing to incomplete reporting of event rates (Darmstadt et al., 2005; Horbar et al., 2001; Kaplan et al., 2011; Kilbride et al., 2003). Although the LOS incidence was reported in these studies, the number of events remained unprovided. The other four excluded studies were omitted because of inconsistent calculation methods for the LOS incidence rates (Balla et al., 2018; Bizzarro et al., 2010; Bloom et al., 2003; Gill et al., 2011). In these studies, the LOS incidence rates were calculated as the number of events per 1,000 patient days, which significantly deviated from the calculation approach adopted by the included studies.

**Figure 2:**
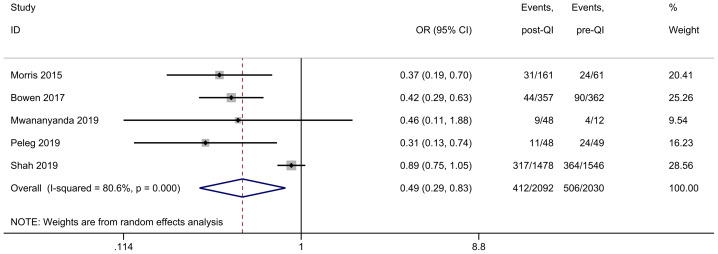
Forest plot from random effects analysis: the rate of LOS in ELBW infants pre- and post-QI. Notes: Morris, Cleary & Soliman, 2015; Bowen et al., 2017; Mwananyanda et al., 2019; Peleg et al., 2019; Shah et al., 2019.

Among the 21 studies included in the meta-analysis, five provided dichotomous data on the incidence of LOS before and after QI in neonates with a birth weight of < 1,000 g or GA < 29 weeks (Bowen et al., 2017; Morris, Cleary & Soliman, 2015; Mwananyanda et al., 2019; Peleg et al., 2019; Shah et al., 2019). Eight NICUs in Australia conducted a QI project over 3 years targeting neonates < 29 weeks gestation (sample size: 719) (Bowen et al., 2017). The project results in a > 50% reduction in bloodstream infection. CHOC Children’s Hospital in the USA provided care in a district unit using quality improvement methods, which improved outcomes in ELBW infants (sample size: 222) (Morris, Cleary & Soliman, 2015). The program resulted in a significant decrease in the hospital-acquired infection rate from 39.3% to 19.4%. University Teaching Hospital in Zambia implemented a two-year QI program with a multi-faceted infection prevention and control bundle. It demonstrated that the rate of LOS was lower in the intervention than the baseline period in ELBW infants (9/48 *vs.* 4/12) (Mwananyanda et al., 2019). The Sheba Medical Center in Israel carried out a comprehensive protocol for preterm infants, in which the LOS rates lowered significantly compared with the controls (11/48 *vs.* 24/49) among preterm infants 28 weeks and under (Peleg et al., 2019). Another improved continuous QI initiative was carried out in 25 tertiary NICUs in Canada for 5 years (sample size: 3,024) (Shah et al., 2019), reporting a decrease of LOS from 24% to 21%. The combined results showed that QI efforts substantially reduce the incidence of LOS in ELBW infants (OR 0.49, 95% CI [0.29–0.83]) (Fig. 2). Given the substantial heterogeneity between studies (*I*^2^ = 80.6%), we identified the multicenter study (Shah et al., 2019) as a potential source of heterogeneity. Excluding that study led to homogeneity among the studies (*I*^2^ = 0.0%), and the meta-analysis result remained stable (Appendix S3).

**Figure 3:**
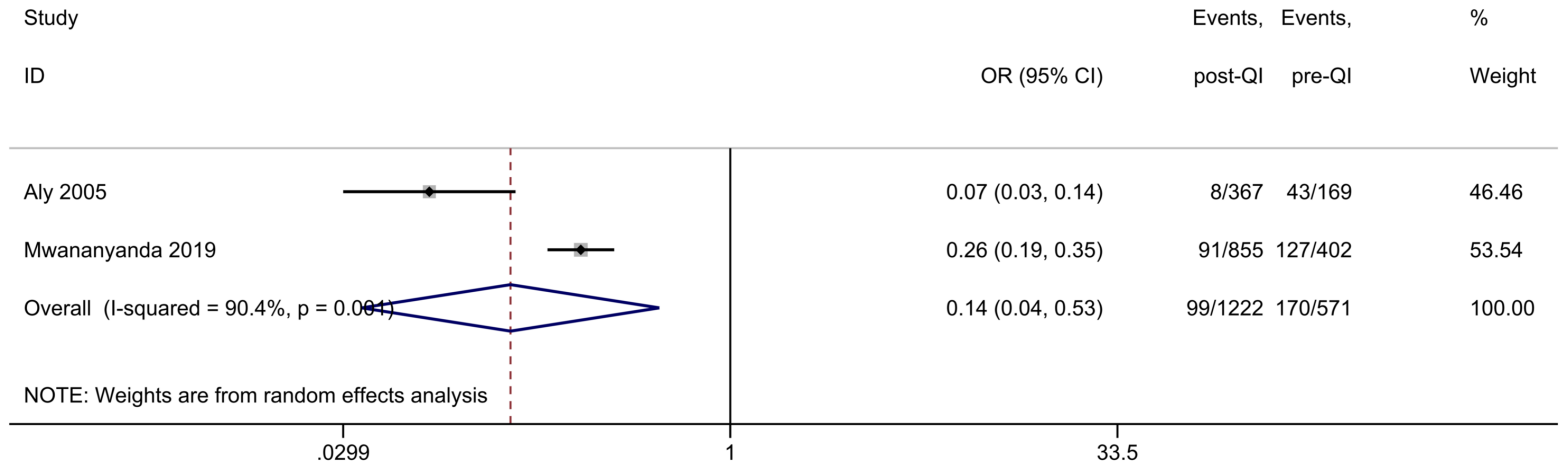
Forest plot from random effects analysis: the rate of LOS in LBW infants pre- and post-QI. Notes: Aly et al., 2005; Mwananyanda et al., 2019.

Two studies provided insights into LOS occurrences among LBW infants with a GA < 37 weeks or birth weight < 2,500 g (Aly et al., 2005; Mwananyanda et al., 2019). The first study involved 536 cases (Aly et al., 2005), revealing that the incidence of LOS dropped from 25.4% to 2.2%. The second study, conducted at the University Teaching Hospital in Zambia (Mwananyanda et al., 2019), included 1,257 cases and reported that the rate of hospital-associated sepsis was lower in the intervention period compared to the baseline (31.6% to 10.6%). A subsequent meta-analysis combining the effect sizes from both studies determined an overall OR = 0.14, 95% CI [0.04−0.53], and *I*^2^ = 90.4%, highlighting a high degree of heterogeneity among the findings (Fig. 3). This high heterogeneity can be attributed to significant differences in the study settings. The second study was conducted in Zambia, a country with limited healthcare resources and technological capabilities compared to the United States, where the first study was carried out. Despite successfully reducing the LOS incidence through quality improvement measures, Zambia’s healthcare system likely faced multiple constraints that prevented it from achieving the same level of improvement as the studies from the USA.

**Figure 4:**
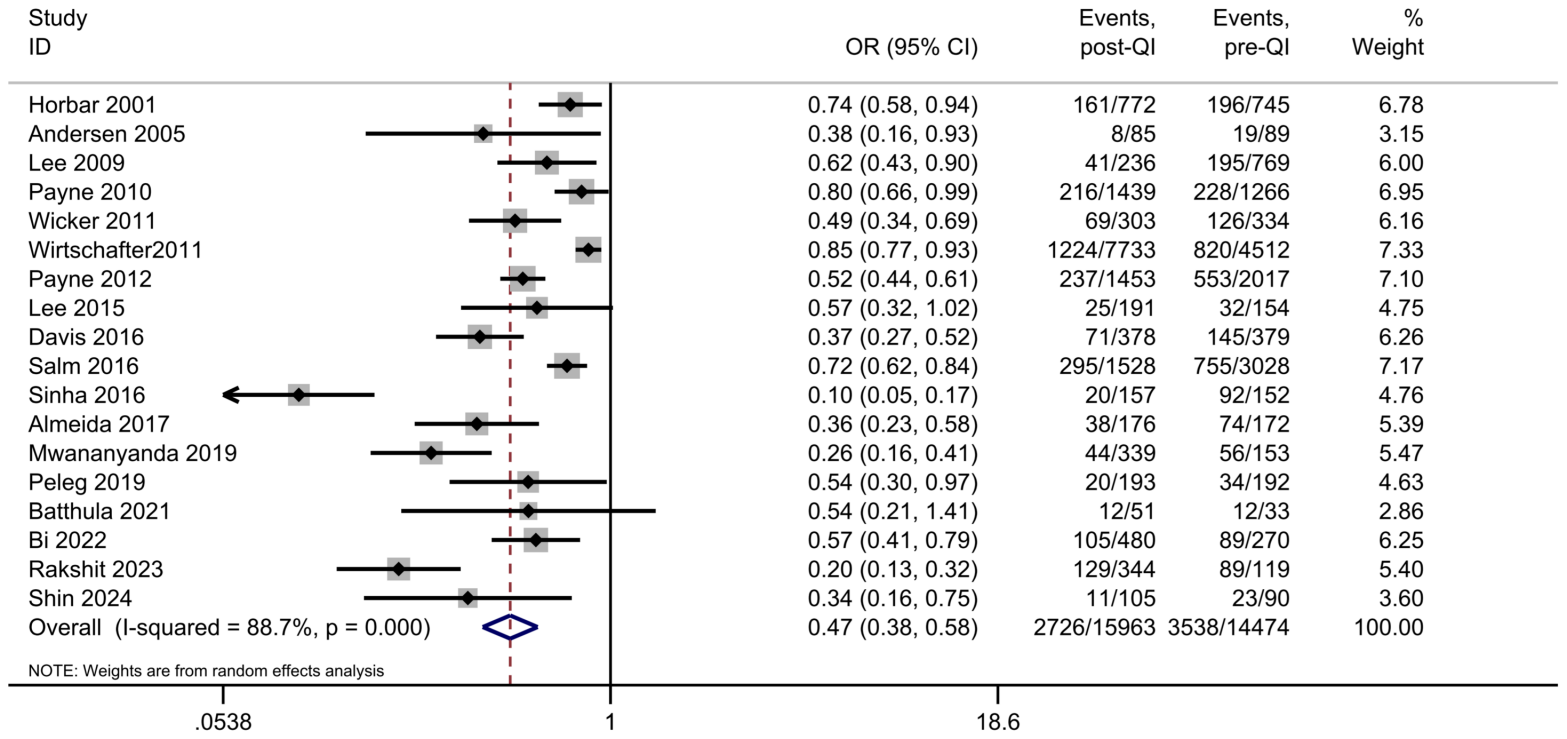
Forest plot from random effects analysis: the rate of LOS in VLBW infants pre- and post-QI. Note: Horbar et al., 2001; Mwananyanda et al., 2019; Payne et al., 2012; Payne et al., 2010; Wicker et al., 2011; Wirtschafter et al., 2011; Andersen et al., 2005; Lee et al., 2009; Batthula, Somnath & Datta, 2021; Rakshit et al., 2023; Davis et al., 2016; Sinha et al., 2016; Bi et al., 2022; Salm et al., 2016; Peleg et al., 2019; Shin et al., 2024; Almeida et al., 2017.

Among the 21 studies included in the meta-analysis, 18 reported on the incidence of LOS in VLBW infants or infants with a GA < 33 weeks (Almeida et al., 2017; Andersen et al., 2005; Batthula, Somnath & Datta, 2021; Bi et al., 2022; Davis et al., 2016; Horbar et al., 2001; Lee et al., 2015; Lee et al., 2009; Mwananyanda et al., 2019; Payne et al., 2012; Payne et al., 2010; Peleg et al., 2019; Rakshit et al., 2023; Salm et al., 2016; Shin et al., 2024; Sinha et al., 2016; Wicker et al., 2011; Wirtschafter et al., 2011). The combined sample size was 30,437, comprising 2,726 participants in the QI intervention group and 3,538 in the control group. Initially, the incidence of LOS ranged from 17.7% to 74.8%. Following the introduction of the QI bundle, this rate decreased significantly to between 9.4% and 37.5%. A notable reduction in LOS was observed across all studies, with the meta-analysis indicating a combined OR = 0.47, 95% CI [0.38–0.58] (Fig. 4). The heterogeneity of the included studies was high: *I*^2^ = 88.7%. However, sensitivity analysis showed a combined OR = 0.47 (95% CI [0.38–0.58]), the lowest estimate of 0.45 after the study by Wirtschafter et al. (2011) was omitted, and the highest estimate of 0.52 after the study by Sinha et al. (2016) was omitted (Appendix S4). Meta-regression analysis did not reveal significant modifiers to the results. Study year Coef. β = 0.55 eβ = 1.74 *p* = 0.12, population size Coef. β = 0.03 eβ = 1.03 *p* = 0.92, clinical setting Coef. β =0.22 eβ = 1.25 *p* = 0.52, study quality score Coef. β = 0.03 eβ = 1.03 *p* = 0.93. Subgroup analysis stratified by the definition of late-onset sepsis demonstrated distinct findings (Appendix S5). Among studies that defined late-onset sepsis using a 48-hour time threshold, the OR for LOS was 0.35 (95% CI [0.19–0.64]), with substantial heterogeneity observed (*I*^2^ = 87.1%). When a 72-hour time threshold was applied, the OR for LOS was 0.50 (95% CI [0.39–0.64]), accompanied by a high degree of heterogeneity (*I*^2^ = 88.9%). In contrast, for studies with an ill-defined time threshold, the OR for LOS was 0.71 (95% CI [0.61–0.82]), and no heterogeneity was detected (*I*^2^ = 0.0%). The stark contrast in heterogeneity across subgroups suggests that inconsistent LOS definitions (*e.g.*, 48 *vs.* 72 h) may contribute to overall heterogeneity. The absence of heterogeneity in ill-defined studies might reflect homogeneous methodological flaws rather than true effect consistency, highlighting the need for standardized time thresholds in future research.

### Publication bias

Due to the limited number of studies on late-onset sepsis in ELBW and LBW infants, which did not exceed ten, a funnel plot was not generated to assess publication bias. For the studies on late-onset sepsis in VLBW infants, asymmetry was assessed by the visual inspection of the funnel plot (Appendix S6). Egger’s regression showed a significant analysis on VLBW infants, *p* < 0.05, indicating publication bias.

## Discussion

This systematic review and meta-analysis of 29 QI studies (21 included in quantitative synthesis, *n* = 29,120 preterm infants) provide robust evidence that QI bundles significantly reduce LOS in premature infants, with a pooled OR of 0.47 (95% CI [0.38–0.58]) for VLBW infants and 0.49 (95% CI [0.29–0.83]) for ELBW infants. These findings align with the global imperative to prioritize prevention in vulnerable neonates, where LOS remains a leading cause of mortality and long-term neurodevelopmental harm (Flannery et al., 2022; Kurul et al., 2023).

### Core components and cross-context efficacy

Across 29 studies, the most frequently cited QI bundle components—multidisciplinary teamwork, staff education, optimized hand hygiene, and central line management—consistently correlated with reduced LOS in diverse settings. These range from high-resource NICUs in the USA and Australia to resource-limited environments in Zambia and India. This consistency highlights the universal importance of foundational infection control practices, which target critical pathophysiological vulnerabilities in preterm infants, such as immature immune systems and compromised skin barriers (Collins, Weitkamp & Wynn, 2018). For instance, structured educational initiatives have been shown to strengthen compliance with hand hygiene and catheter care protocols, directly addressing key pathways for pathogen transmission (Rakshit et al., 2023). Multidisciplinary collaboration (25/29 studies) likely enhances care coordination by integrating expertise from neonatologists, infection control nurses, and dietitians, forming a “prevention–monitoring–intervention” cycle. At CHOC Children’s Hospital in the USA, such collaboration reduced LOS in ELBW infants from 39.3% to 19.4% (Morris, Cleary & Soliman, 2015), exemplifying the value of systemic intervention.

In seven of the 29 studies, early enteral feeding with human milk is recognized as a relevant intervention. Its potential mechanisms include maintaining intestinal barrier integrity and modulating gut microbiota, which may help reduce bacterial translocation (Taylor, 2019). Chitale et al. (2022) found that the link between early enteral feeding and reduced mortality and sepsis is clinically reasonable. Notably, delayed enteral feeding often requires parenteral nutrition or intravenous fluids, which increase the risks of bloodstream infections and metabolic problems—factors that may account for the lower rate of LOS in the early-feeding groups. A study in Italy showed that human milk feeding (including mother’s milk and donor human milk) reduced the risk of LOS by 22% to 66% in VLBW infants (Quitadamo et al., 2025), highlighting the interaction between nutrition and immune protection in preterm infants.

### Heterogeneity and methodological considerations

Substantial heterogeneity in LOS definitions (*e.g.*, 48 *vs.* 72 h post-birth) and diagnostic criteria complicated data synthesis, underscoring the need for standardized reporting. Studies using a 72-hour threshold (55.2% of included studies) showed an OR of 0.50 (95% CI [0.39–0.64]), whereas those with ill-defined thresholds had weaker effects (OR = 0.71), emphasizing the impact of uniform case definitions on comparability (Fleischmann-Struzek et al., 2018). Methodological limitations, such as non-randomized before-and-after designs, introduced risks of bias from secular trends (*e.g.*, improved antibiotic stewardship) or the Hawthorne effect (Vaisman et al., 2020). However, the high quality of most studies (27/29 classified as high *via* QI-MQCS) and consistent effect directions across subgroups strengthen validity.

Sample size disparities (68–12,245 participants) also influenced heterogeneity. For instance, a single-center study in Zambia (*n* = 1,257) reported an extreme OR of 0.14, possibly due to a small sample size and limited interventions (Mwananyanda et al., 2019). In contrast, a Canadian multicenter study (*n* = 3,024) with standardized protocols showed an OR of 0.71 (Shah et al., 2019). Sensitivity analyses excluding outlier studies stabilized results (*e.g.*, ELBW subgroup *I*^2^ decreased from 80.6% to 0%, Appendix S3), supporting the robustness of QI effects. Egger’s test for VLBW infants suggested publication bias (*p* < 0.05, Appendix S6), which may overestimate the observed effect sizes. This highlights the need for pre-registered study protocols and transparent reporting of negative results to mitigate selective publication in future research.

### Implementation challenges and equity implications

While QI bundles were effective globally, resource disparities profoundly shaped outcomes. Middle-income countries like India and Zambia achieved significant reductions in LOS, for example, a 36.4% to 23.5% decrease in India (Batthula, Somnath & Datta, 2021) and a 31.6% to 10.6% decrease in Zambia (Mwananyanda et al., 2019). Despite these achievements, scaling up advanced interventions remains a challenge. Infrastructure limitations in these regions impede the full implementation of QI strategies, underscoring how resource availability dictates such initiatives’ reach and long-term viability.

Examining the NICU environment in the USA reveals equity implications. Research by Horbar et al. (2019) highlights the complex relationship between social factors, geography, and NICU quality. Infants from socially disadvantaged groups disproportionately receive care in NICUs with lower quality scores, while those from more privileged socioeconomic backgrounds are more likely to be treated in higher-performing units. This disparity aligns with broader regional differences; for instance, the resource-constrained Mountain region has a weighted mean NICU quality score of −0.69, starkly contrasting with the 0.85 score in the well-resourced Pacific region. These patterns indicate how systemic factors, specifically the unequal distribution of healthcare resources tied to socioeconomic and geographic contexts, contribute to unequal access to care. Resolving these discrepancies is essential for enhancing clinical outcomes and ensuring fairness in healthcare provision, underscoring the urgent need to address the structural issues perpetuating these disparities in neonatal care.

### Future directions for research and practice

The dominance of studies from high-income countries (22 out of 29) highlights an urgent need for research in regions with the highest sepsis burdens, where incidence rates can be up to 40 times higher (Fleischmann-Struzek et al., 2018). Future QI initiatives should adopt context-adaptive designs, such as substituting chlorhexidine with alcohol-based hand sanitizers in resource-limited settings while rigorously assessing scalability. Methodologies like stepped-wedge or pragmatic randomized controlled trials are recommended to mitigate time-trend biases. Concurrently, the escalating threat of multidrug resistance demands urgent intervention, exemplified by a Brazilian NICU study where Enterobacterales isolates showed 25% cefepime resistance. In comparison, *Acinetobacter baumannii* exhibited resistance rates of 36% (cefepime), 27% (amikacin), and 31% (meropenem) (Freitas et al., 2023).

These challenges highlight the imperative to integrate antibiotic stewardship into QI frameworks. A Chinese Level 4 NICU study demonstrated this approach’s efficacy: implementing a two-year stewardship program combining local microbiological data with de-escalation protocols—including prompt antibiotic discontinuation for ruled-out sepsis and evidence-based pneumonia treatment durations—reduced multidrug-resistant bacterial infections from 67.2% to 48.9% (Ren et al., 2023). Future work should standardize definitions (*e.g.*, LOS defined as ≥72 h with positive blood/CSF cultures) to strengthen cross-study comparability and systematically report pathogen profiles.

### Strengths and limitations

This meta-analysis encompasses both notable strengths and limitations that merit careful consideration. As the pioneering quantitative assessment of QI bundles aimed at reducing LOS among preterm infants at birth, it effectively addresses a critical void in the existing research landscape. Through an exhaustive systematic review process, we gathered and synthesized relevant studies, providing an in-depth understanding of QI bundle components while quantitatively evaluating LOS incidence rates across LBW, VLBW, and ELBW infants. The fact that all included studies met moderate to high-quality standards, as verified by the QI-MQCS tool, adds significant weight to the reliability of our findings.

However, several challenges emerged during the analysis. The considerable variation in how LOS was defined and diagnosed across studies posed difficulties in synthesizing the data, highlighting the urgent need for standardized reporting practices. Methodological limitations, such as using non-randomized before-and-after study designs, introduced potential biases stemming from secular trends and the Hawthorne effect. Discrepancies in sample sizes across different studies further contributed to heterogeneity, and the detected publication bias among VLBW infants may have led to an overestimation of the effect sizes. Additionally, the wide disparities in global resources and structural inequalities in neonatal care settings restrict the generalizability of our results. Addressing these limitations will be crucial for advancing future research on the effectiveness of QI bundles for preterm infants.

## Conclusions

This systematic review confirms that quality improvement bundles effectively reduce late-onset sepsis in preterm infants, with consistent benefits across very low and extremely low birth weight groups. The core strategies, which include multidisciplinary teamwork, optimized hand hygiene, standardized central line care, and staff education, address both the biological vulnerabilities of preterm infants and the risks of medical care in NICUs. Variations in sepsis definitions highlight the need for standardized diagnostic criteria to improve comparability, while resource constraints emphasize the importance of adapting these approaches for low-resource settings—a need grounded in the evidence from our review and broader healthcare principles. Clinically, these evidence-based bundles should be integrated into routine neonatal care globally to reduce sepsis-related complications and mortality. To ensure global access to these proven preventive measures, key priorities include establishing uniform outcome reporting, incorporating antibiotic stewardship, and addressing healthcare inequities, all of which leverage the findings of this review.

## Supplemental Information

10.7717/peerj.20530/supp-1 Supplemental Information 1 Search strategy

10.7717/peerj.20530/supp-2 Supplemental Information 2 Quality and risk of bias assessment

10.7717/peerj.20530/supp-3 Supplemental Information 3 Forest plot of LOS in ELBW infants excluded *Shah 2019*

10.7717/peerj.20530/supp-4 Supplemental Information 4 Sensitivity analysis for studies among VLBW infants

10.7717/peerj.20530/supp-5 Supplemental Information 5 Forest plot of LOS among VLBW infants stratified by the definition of LOS (time window)

10.7717/peerj.20530/supp-6 Supplemental Information 6 Funnel plot for LOS in VLBW infants

10.7717/peerj.20530/supp-7 Supplemental Information 7 Excluded full-text articles with reasons

10.7717/peerj.20530/supp-8 Supplemental Information 8 PRISMA checklist

10.7717/peerj.20530/supp-9 Supplemental Information 9 Rationale

10.7717/peerj.20530/supp-10 Supplemental Information 10 Raw data Events of late-onset sepsis pre- and post-QI.

## Additional Information and Declarations

### Data Availability

The following information was supplied regarding data availability: The raw data are available in the Supplementary Files.
