# Supplementary material for: Quality improvement interventions to prevent late-onset sepsis in premature infants: a systematic review and meta-analysis
Source: PeerJ. 2026 Jan 2;14:e20530. doi: 10.7717/peerj.20530 (PMC12767489; doi:10.7717/peerj.20530)
Supplement: Supplemental Information 1 [file peerj-14-20530-s001.docx]

**Search strategies**

**Pubmed**

| #1 | ((((((((((bloodstream infection[Title/Abstract]) OR (hospital acquired infection[Title/Abstract])) OR (nosocomial infection[Title/Abstract])) OR (healthcare acquired infection[Title/Abstract])) OR (hospital-acquired infection[Title/Abstract])) OR (healthcare-acquired infection[Title/Abstract])) OR (late-onset sepsis[Title/Abstract])) OR (late-onset neonatal sepsis[Title/Abstract])) OR (neonatal sepsis[Title/Abstract])) OR (sepsis, neonatal[Title/Abstract])) OR (("Sepsis"[Mesh] OR "Neonatal Sepsis"[Mesh] OR "Systemic Inflammatory Response Syndrome"[Mesh]) OR "Cross Infection"[Mesh]) | 229,714 |
| --- | --- | --- |
| #2 | ((((((((quality improvement[Title/Abstract]) OR (strategies[Title/Abstract])) OR (strategy[Title/Abstract])) OR (prevention[Title/Abstract])) OR (measures[Title/Abstract])) OR (care bundles[Title/Abstract])) OR (interventions[Title/Abstract])) OR (initiative[Title/Abstract])) OR ("Quality Improvement"[Mesh]) | 3,947,633 |
| #3 | (("Infant, Low Birth Weight"[Mesh] OR "Infant, Extremely Low Birth Weight"[Mesh] OR "Infant, Very Low Birth Weight"[Mesh]) OR ( "Premature Birth"[Mesh] OR "Infant, Extremely Premature"[Mesh] OR "Infant, Premature"[Mesh] OR "Infant, Premature, Diseases"[Mesh] )) OR (((((((((((preterm[Title/Abstract]) OR (premature[Title/Abstract])) OR (pre term[Title/Abstract])) OR (prematurity[Title/Abstract])) OR (low birth weight[Title/Abstract])) OR (low birthweight[Title/Abstract])) OR (very low birth weight[Title/Abstract])) OR (extremely low birth weight[Title/Abstract])) OR (LBW[Title/Abstract])) OR (VLBW[Title/Abstract])) OR (ELBW[Title/Abstract])) | 317,637 |
| #1 AND #2 AND #3 | (((("Infant, Low Birth Weight"[Mesh] OR "Infant, Extremely Low Birth Weight"[Mesh] OR "Infant, Very Low Birth Weight"[Mesh]) OR ( "Premature Birth"[Mesh] OR "Infant, Extremely Premature"[Mesh] OR "Infant, Premature"[Mesh] OR "Infant, Premature, Diseases"[Mesh] )) OR (((((((((((preterm[Title/Abstract]) OR (premature[Title/Abstract])) OR (pre term[Title/Abstract])) OR (prematurity[Title/Abstract])) OR (low birth weight[Title/Abstract])) OR (low birthweight[Title/Abstract])) OR (very low birth weight[Title/Abstract])) OR (extremely low birth weight[Title/Abstract])) OR (LBW[Title/Abstract])) OR (VLBW[Title/Abstract])) OR (ELBW[Title/Abstract]))) AND (((((((((quality improvement[Title/Abstract]) OR (strategies[Title/Abstract])) OR (strategy[Title/Abstract])) OR (prevention[Title/Abstract])) OR (measures[Title/Abstract])) OR (care bundles[Title/Abstract])) OR (interventions[Title/Abstract])) OR (initiative[Title/Abstract])) OR ("Quality Improvement"[Mesh]))) AND (((((((((((bloodstream infection[Title/Abstract]) OR (hospital acquired infection[Title/Abstract])) OR (nosocomial infection[Title/Abstract])) OR (healthcare acquired infection[Title/Abstract])) OR (hospital-acquired infection[Title/Abstract])) OR (healthcare-acquired infection[Title/Abstract])) OR (late-onset sepsis[Title/Abstract])) OR (late-onset neonatal sepsis[Title/Abstract])) OR (neonatal sepsis[Title/Abstract])) OR (sepsis, neonatal[Title/Abstract])) OR (("Sepsis"[Mesh] OR "Neonatal Sepsis"[Mesh] OR "Systemic Inflammatory Response Syndrome"[Mesh]) OR "Cross Infection"[Mesh])) | 1,776 |

**Embase**

| **No.** | **Query** | **Results** |
| --- | --- | --- |
| **#1** | **'prematurity'/exp OR 'newborn'/exp OR 'low birth weight'/exp OR 'very low birth weight'/exp OR 'extremely low birth weight'/exp** | **833834** |
| **#2** | **'late onset sepsis'/exp OR 'cross infection'/exp OR 'healthcare associated infection'/exp OR 'hospital infection'/exp OR 'newborn sepsis'/exp OR 'bloodstream infection'/exp OR 'nosocomial infection'/exp** | **356505** |
| **#3** | **'total quality management'/exp OR 'quality improvement study'/exp OR 'strategy*':ab,ti OR 'strategies*':ab,ti OR 'management':ab,ti OR 'prevention':ab,ti OR 'bundles':ab,ti OR 'quality improvement':ab,ti OR 'measures':ab,ti** | **6090321** |
| **#4** | **#1 AND #2 AND #3** | **5821** |

**The Cochrane Library**

ID        Search        Hits

#1        MeSH descriptor: [Infant, Premature] explode all trees        5665

#2        MeSH descriptor: [Infant, Very Low Birth Weight] explode all trees        1344

#3        MeSH descriptor: [Infant, Extremely Low Birth Weight] explode all trees        176

#4        MeSH descriptor: [Quality Improvement] explode all trees        1247

#5        MeSH descriptor: [Cross Infection] explode all trees        2217

#6        MeSH descriptor: [Neonatal Sepsis] explode all trees        142

#7        ("preterm" OR "premature" OR "very low birth weight" OR "extremely low birth weight"):ti,ab,kw (Word variations have been searched)        36714

#8        ("late-onset sepsis" OR "neonatal sepsis" OR "nosocomial infection" OR "cross infection" OR "hospital infection" OR "healthcare acquired infection" OR "bloodstream infection"):ti,ab,kw (Word variations have been searched)        5219

#9        ("quality improvement" OR "management" OR "prevention" OR "strategies" OR "bundles" OR "measures"):ti,ab,kw (Word variations have been searched)        974493

#10        #1 OR #2 OR #3 OR #7        36714

#11        #4 OR #9        974498

#12        #5 OR #6 OR #8        5788

#13        #10 AND #11 AND #12        567

**Web of Science**

| # | Search Query | Database | Results |
| --- | --- | --- | --- |
| 1 | TS=("preterm" OR "premature" OR "very low birth weight" OR "extremely low birth weight") | Web of Science Core Collection | 298086 |
| 2 | TS=("late-onset sepsis" OR "neonatal sepsis" OR "nosocomial infection" OR "hospital infeciton" OR "healthcare acquired infection" OR "cross infection" OR "bloodstream infection") | Web of Science Core Collection | 28687 |
| 3 | TS=("quality improvement" OR "measures" OR "management" OR "strategies" OR "bundles" OR "prevention" OR "interventions") | Web of Science Core Collection | 7967174 |
| 4 | #1 AND #2 AND #3 | Web of Science Core Collection | 1541 |
