# Supplementary figures and images for: Quality improvement interventions to prevent late-onset sepsis in premature infants: a systematic review and meta-analysis
Source: PeerJ. 2026 Jan 2;14:e20530. doi: 10.7717/peerj.20530 (PMC12767489; doi:10.7717/peerj.20530)

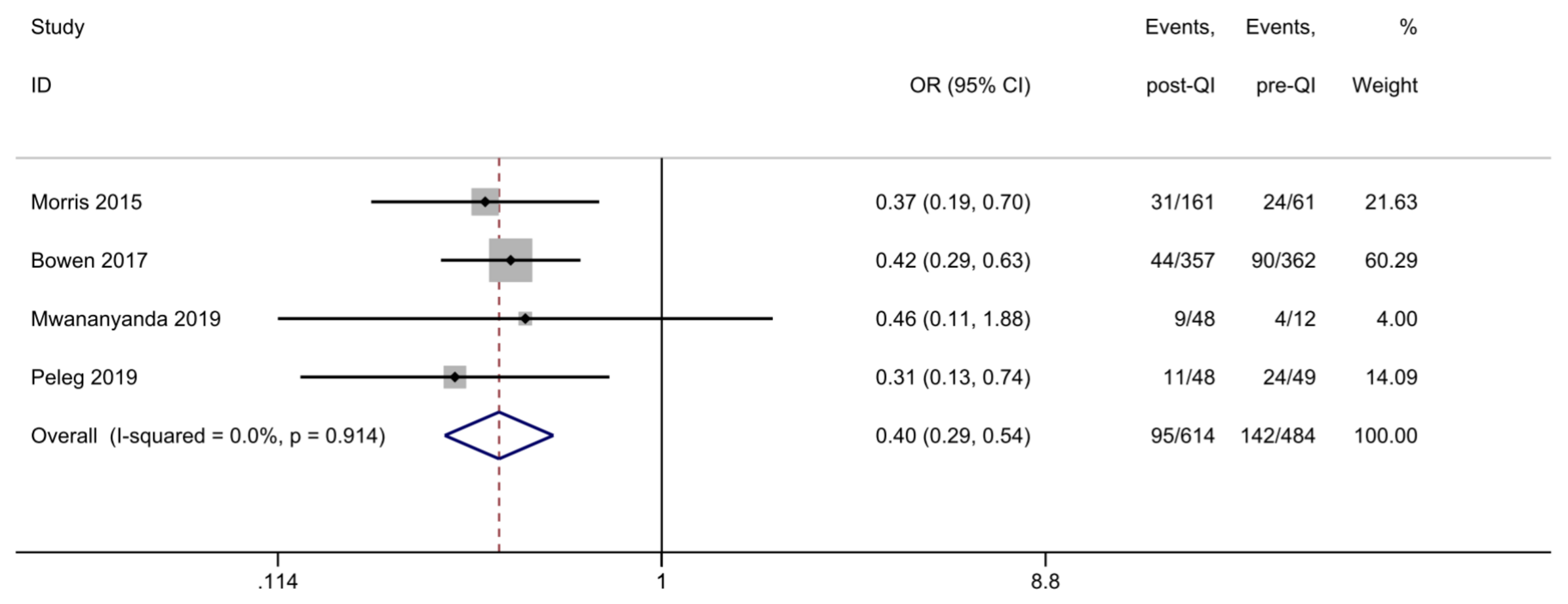

Supplement: Supplemental Information 3 [file peerj-14-20530-s003.png]

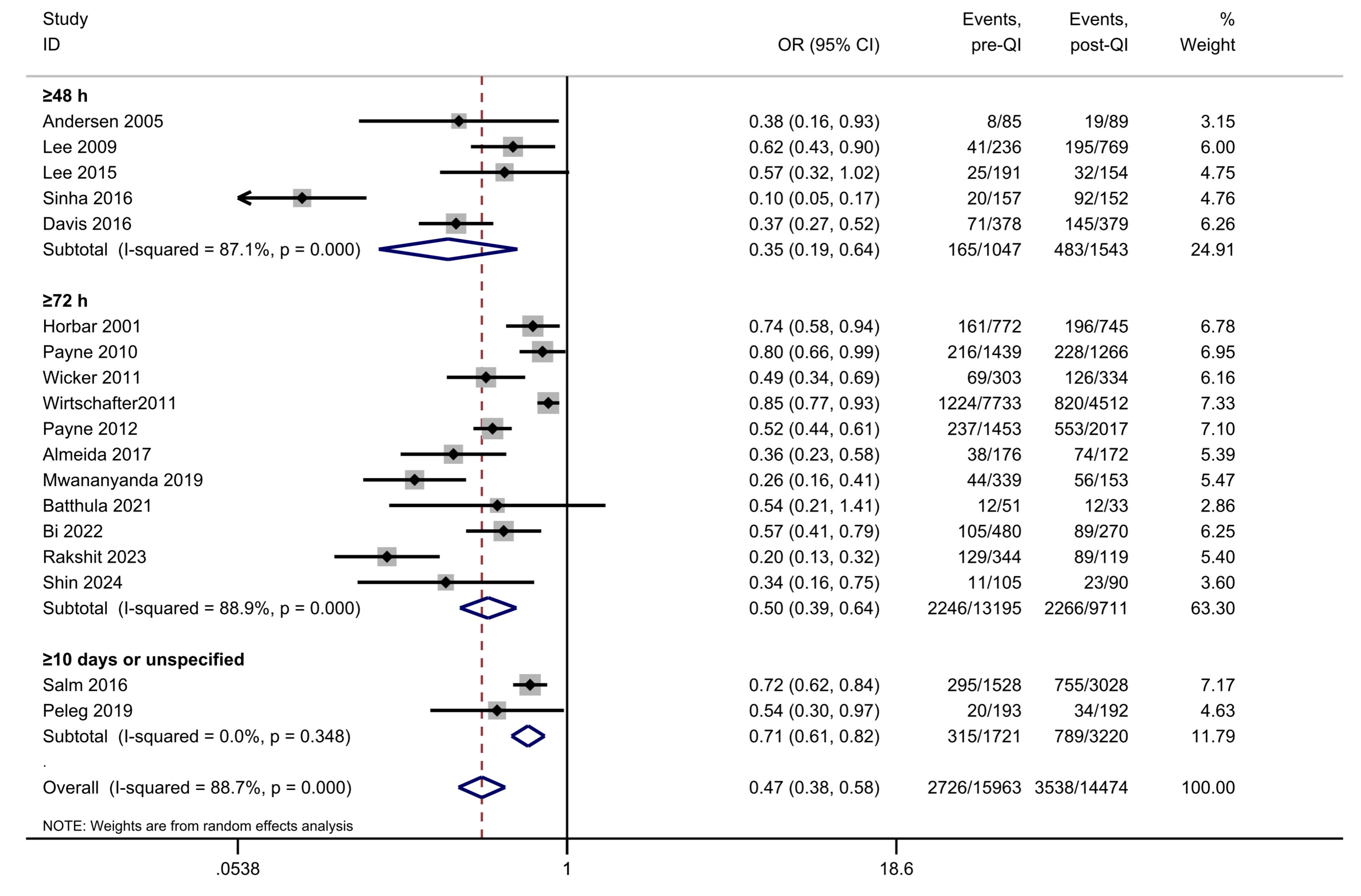

Supplement: Supplemental Information 5 [file peerj-14-20530-s005.png]

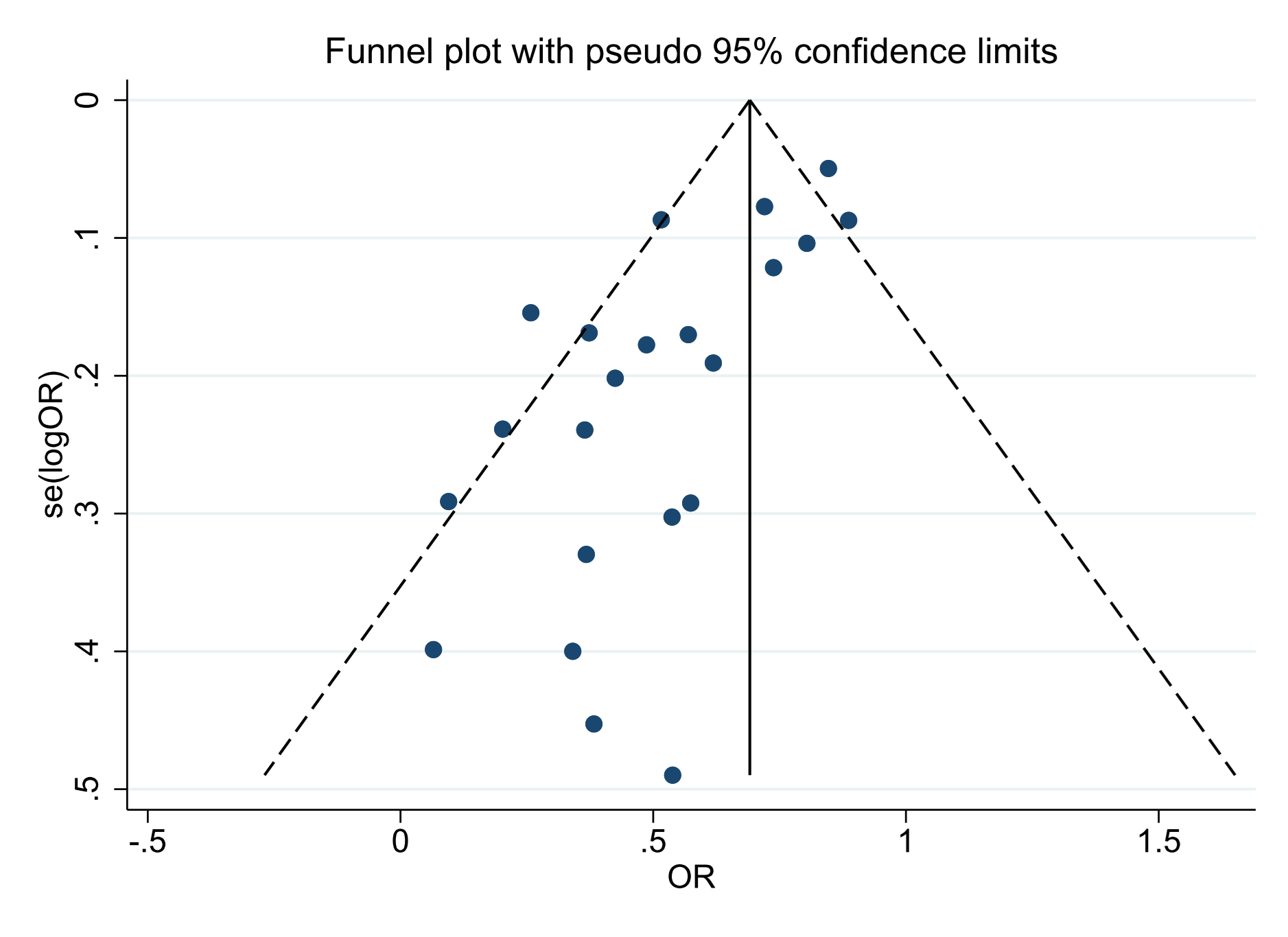

Supplement: Supplemental Information 6 [file peerj-14-20530-s006.png]
