# Supplementary material for: Quality improvement interventions to prevent late-onset sepsis in premature infants: a systematic review and meta-analysis
Source: PeerJ. 2026 Jan 2;14:e20530. doi: 10.7717/peerj.20530 (PMC12767489; doi:10.7717/peerj.20530)
Supplement: Supplemental Information 4 [file peerj-14-20530-s004.docx]

**Appendix 5** Sensitivity analysis for studies among VLBW infants.

------------------------------------------------------------------------------

Study omitted | Estimate [95% Conf. Interval]

-------------------+----------------------------------------------------------

Andersen 2005 | .47218042 .38246457 .58294119

Wicker 2011 | .4673978 .37652406 .58020383

Wirtschafter2011 | .44746952 .36038349 .55559974

Almeida 2017 | .47624341 .38540518 .58849179

Peleg 2019 | .46565771 .37607412 .57658076

Batthula 2021 | .4669799 .37809953 .57675349

Bi 2022 | .46212012 .37159429 .57469937

Rakshit 2023 | .49595678 .4064127 .60522993

Shin 2024 | .47485418 .38468843 .58615357

Horbar 2001 | .45226442 .3621483 .56480483

Lee 2009 | .46001962 .37016889 .57167975

Payne 2010 | .44892226 .35915045 .56113308

Payne 2012 | .46337374 .37001283 .58029129

Lee 2015 | .46397247 .37457623 .574704

Davis 2016 | .47712816 .38634605 .58924189

Salm 2016 | .45026703 .35641688 .56882939

Mwananyanda 2019 | .48769817 .39725114 .59873838

Sinha 2016 | .51506203 .42876896 .61872224

-------------------+----------------------------------------------------------

Combined | .46913675 .38138121 .57708477

------------------------------------------------------------------------------
