# Supplementary material for: Quality improvement interventions to prevent late-onset sepsis in premature infants: a systematic review and meta-analysis
Source: PeerJ. 2026 Jan 2;14:e20530. doi: 10.7717/peerj.20530 (PMC12767489; doi:10.7717/peerj.20530)
Supplement: Supplemental Information 7 [file peerj-14-20530-s007.docx]

**Table S1** Excluded full-text articles with reasons.

| **Study** | **Reason** |
| --- | --- |
| (Agarwal et al., 2024; Alp et al., 2019; Cavicchiolo et al., 2016; Dramowski et al., 2023; Johnson et al., 2021; Kallimath et al., 2024; Koch et al., 2015; Landre-Peigne et al., 2011; Neill et al., 2016; Patodia et al., 2021; Pharande et al., 2014; Shettigar et al., 2021) | Mixed preterm and term populations, no separable preterm data 12 |
| (Lee et al., 2014b) | Same study population 1 |
| (Abreu et al., 2011; Acevedo et al., 2012; Adams & Bassler, 2019; Bowen et al., 2016; Cao et al., 2019; Chaudhari et al., 2017; Davis et al., 2014; Fang et al., 2014; Fitzgerald et al., 2022; González et al., 2014; Hang et al., 2015; Huang et al., 2015; Kawagoe et al., 2009; Kessler et al., 2011; Mazi et al., 2019; McKechnie & Sethi, 2016; Mohamed Cassim et al., 2015; Murray et al., 2024; Murthy et al., 2014; Nyantakyi et al., 2025; Okwujiako et al., 2024; Oved et al., 2009; Pammi & Weisman, 2015; Perkins et al., 2022; Polin et al., 2012; Powers & Wirtschafter, 2010; Qadir et al., 2015; Ramakrishnan et al., 2015; Rani et al., 2020; Resende et al., 2011; Sanchez, 2010; Scott, 2016; Simeoni, 2011; Wanigasekara et al., 2023; Yasmeen et al., 2011; Zaka et al., 2018) | Only conference abstracts available 36 |
| (Adams-Chapman & Stoll, 2002; Alcock et al., 2017; Ks et al., 2021; Ran et al., 2019; Rohsiswatmo et al., 2014; Saiman, 2006; Schulman et al., 2009; Short, 2019) | Not a quality improvement study 8 |
| (Ambreen et al., 2022; Ren et al., 2023; Singh et al., 2024; Zini et al., 2024) | Quality improvement goal unassociated with LOS reduction 4 |
| (Al Bizri et al., 2023; Balachander et al., 2020; Bharadwaj et al., 2019; Bissainte-Zelbin et al., 2025; Ceballos et al., 2013; Chen et al., 2025; Deshommes et al., 2021; Ferorelli et al., 2024; Grover et al., 2015; Hawes & Lee, 2018; Huang et al., 2019; Hussain et al., 2021; Jain et al., 2024; Khurana et al., 2018; Lee et al., 2014a; Nankervis et al., 2010; Resende et al., 2015; Rogers et al., 2010; Savage et al., 2018; Sharma et al., 2020; Shepherd et al., 2015; Steiner et al., 2015; Thor et al., 2010; Wheeler et al., 2011) | Lack of a clear definition of LOS, or the reported outcomes were wrong or non-specific 26 |

**References**

**Abreu M, Kawagoe JY, De Menezes FG, Forno CB, Dos Santos Cardoso MF, Dornaus MFP, Warth AN, Deutsch AD, Correa L, and Abramovici S**. **2011**. Continuous quality improvement program on healthcare-associated infections in a neonatal intensive care unit: A 16-year experience. *Am J Infect Control* **39(5)**:E84-E85. DOI 10.1016/j.ajic.2011.04.156.

**Acevedo CD, Abate HJ, Rosaenz AM, Falaschi A, Melonari P, Rosaenz L, Gerhardt EL, Guimera D, Johnson KM, and Caniza M**. **2012**. Infection prevention and control program in a public pediatric hospital in Argentina: Opportunities for improvement. *Am J Infect Control* **40(5)**:e108. DOI 10.1016/j.ajic.2012.04.186.

**Adams-Chapman I, and Stoll BJ**. **2002**. Prevention of nosocomial infections in the neonatal intensive care unit. *Curr Opin Pediatr* **14(2)**:157-164. DOI 10.1097/00008480-200204000-00003.

**Adams M, and Bassler D**. **2019**. Practice variations and rates of late onset sepsis and necrotizing enterocolitis in very preterm born infants, a review. *Transl Pediatr* **8(3)**:212-226. DOI 10.21037/tp.2019.07.02.

**Agarwal N, Anand R, Jindal A, Varghese AR, Gajjala C, Ryavanki SP, and Singh G**. **2024**. A Hybrid Form of Telemedicine and Quality Improvement: A Unique Way to Extend Intensive Care Services to Neonates. *Indian J Pediatr*. DOI 10.1007/s12098-024-05200-3.

**Al Bizri A, Hanna Wakim R, Obeid A, Daaboul T, Charafeddine L, Mounla N, Nakad P, and Yunis K**. **2023**. A Quality improvement initiative to reduce central line-associated bloodstream infections in a neonatal intensive care unit in a low-and-middle-income country. *BMJ Open Qual* **12(2)**. DOI 10.1136/bmjoq-2022-002129.

**Alcock G, Liley HG, Cooke L, and Gray PH**. **2017**. Prevention of neonatal late-onset sepsis: a randomised controlled trial. *BMC Pediatr* **17(1)**:98. DOI 10.1186/s12887-017-0855-3.

**Alp E, Cookson B, Erdem H, and Rello J**. **2019**. Infection control bundles in intensive care: an international cross-sectional survey in low- and middle-income countries. *J Hosp Infect* **101(3)**:248-256. DOI 10.1016/j.jhin.2018.07.022.

**Ambreen G, Kumar V, Ali SR, Jiwani U, Khowaja W, Hussain AS, Hussain K, Raza SS, Rizvi A, Ansari U, Ahmad K, Demas S, and Ariff S**. **2022**. Impact of a standardised parenteral nutrition protocol: a quality improvement experience from a NICU of a developing country. *Arch Dis Child* **107(4)**:381-386. DOI 10.1136/archdischild-2021-321552.

**Balachander B, Rajesh D, Pinhero CL, Paul S, Stevens S, and Rao S**. **2020**. Response Measures to Infection Outbreaks During the Second Year of Sustenance Phase of Infection Control Quality Improvement. *Indian J Pediatr* **87(5)**:333-338. DOI 10.1007/s12098-020-03201-6.

**Bharadwaj S, Ho SK, Khong KC, Seet A, Yeo KC, Chan XY, Wong LL, Karlin RB, Chan DK, and Ling ML**. **2019**. Eliminating MRSA transmission in a tertiary neonatal unit–A quality improvement initiative. *Am J Infect Control* **47(11)**:1329-1335. DOI 10.1016/j.ajic.2019.06.001.

**Bissainte-Zelbin V, Durandy A, Lecoq L, Wachter PY, Bennour O, Micklethwait F, Boileau P, and Motte-Signoret E**. **2025**. Morbidity-free survival in extremely premature infants after changes of clinical practices according to evidence-based guidelines: a quality improvement uncontrolled before–after study in a neonatal intensive care unit. *Eur J Pediatr* **184(1)**. DOI 10.1007/s00431-024-05842-7.

**Bowen JR, Callander I, Richards R, and Lindrea KB**. **2016**. Decreasing infection in neonatal intensive care units through quality improvement. *J Paediatr Child Health* **52**:79. DOI 10.1111/jpc.13194.

**Cao Y, Jiang SY, and Zhou Q**. **2019**. Introducing evidence-based practice improvement in Chinese neonatal intensive care units. *Transl Pediatr* **8(3)**:257-261. DOI 10.21037/tp.2019.07.07.

**Cavicchiolo ME, Lanzoni P, Wingi MO, Pizzol D, Daverio M, Da Dalt L, Putoto G, and Trevisanuto D**. **2016**. Reduced neonatal mortality in a regional hospital in Mozambique linked to a Quality Improvement intervention. *BMC Pregnancy Childbirth* **16(1)**. DOI 10.1186/s12884-016-1170-y.

**Ceballos K, Waterman K, Hulett T, and Makic MBF**. **2013**. Nurse-driven quality improvement interventions to reduce hospital-acquired infection in the NICU. *Advances in Neonatal Care* **13(3)**:154-163. DOI 10.1097/ANC.0b013e318285fe70.

**Chaudhari T, Panda M, Fletcher AA, Smith J, and Carlisle H**. **2017**. Central line associatd blood stream infections (CLABSI) in nicu following introduction of a central line bundle. *J Paediatr Child Health* **53**:17. DOI 10.1111/jpc.13494_43.

**Chen H, Miao Z, Cai Y, and Dong L**. **2025**. Effectiveness of Meticulous Management in Reducing Hospital-Acquired Infections in Neonatal Wards: A Prospective Analysis. *Alternative therapies in health and medicine* **31(1)**:78-81. DOI

**Davis JW, Jary S, Cairns PA, Harding D, and Luyt K**. **2014**. Better cognitive outcomes for Very Low Birth Weight Infants after implementation of a sepsis reduction care bundle. *Arch Dis Child* **99**:A202. DOI 10.1136/archdischild-2014-306237.463.

**Deshommes T, Nagel C, Tucker R, Dorcélus L, Gautier J, Koster MP, and Lechner BE**. **2021**. A Quality Improvement Initiative to Increase Hand Hygiene Awareness and Compliance in a Neonatal Intensive Care Unit in Haiti. *J Trop Pediatr* **67(3)**. DOI 10.1093/tropej/fmaa029.

**Dramowski A, Erasmus LM, Aucamp M, Fataar A, Cotton MF, Coffin SE, Bekker A, and Whitelaw AC**. **2023**. SafeHANDS: A Multimodal Hand Hygiene Intervention in a Resource-Limited Neonatal Unit. *Trop Med Infect Dis* **8(1)**. DOI 10.3390/tropicalmed8010027.

**Fang S, Longstaff V, Vanderpool E, Husain S, Claxton A, and Millar M**. **2014**. Sustainable reduction of positive blood cultures in a tertiary neonatal intensive care unit: Impact of infection prevention and control measures over a 5 year period. *Arch Dis Child* **99**:A424-A425. DOI 10.1136/archdischild-2014-307384.1179.

**Ferorelli D, Goffredo VM, Graziano E, Mastrapasqua M, Telegrafo M, Vinci A, Visci P, Benevento M, Zotti F, Foglianese A, Panza R, Solarino B, Dell’Erba A, and Laforgia N**. **2024**. Quality improvement in neonatal care through enhanced patient safety and clinical risk management: a before-and-after study about neonatal sepsis. *Front Med (Lausanne)* **11**. DOI 10.3389/fmed.2024.1430853.

**Fitzgerald FC, Zingg W, Chimhini G, Chimhuya S, Wittmann S, Brotherton H, Olaru ID, Neal SR, Russell N, da Silva ARA, Sharland M, Seale AC, Cotton MF, Coffin S, and Dramowski A**. **2022**. The Impact of Interventions to Prevent Neonatal Healthcare-associated Infections in Low- and Middle-income Countries: A Systematic Review. *Pediatric Infectious Disease Journal* **41(3S)**:S26-S35. DOI 10.1097/INF.0000000000003320.

**González M, Gómez C, Rodríguez MA, Espinosa MG, Sánchez JE, Sánchez T, and Salguero E**. **2014**. Effectiveness of a multimodal intervention on the prevention of nosocomial bloodstream infections in a tertiary neonatal intensive care unit. *Journal of Maternal-Fetal and Neonatal Medicine* **27**:159-160. DOI 10.3109/14767058.2014.924236.

**Grover TR, Pallotto EK, Brozanski B, Piazza AJ, Chuo J, Moran S, McClead R, Mingrone T, Morelli L, and Smith JR**. **2015**. Interdisciplinary teamwork and the power of a quality improvement collaborative in tertiary neonatal intensive care units. *J Perinat Neonatal Nurs* **29(2)**:179-186. DOI 10.1097/JPN.0000000000000102.

**Hang PT, Hang TTT, Anh DPP, Zingg W, and Pittet D**. **2015**. Effectiveness after 5 years of the who hand hygiene promotion strategy to reduce health-care-associated infections at Hung Vuong Hospital, Vietnam. *Antimicrob Resist Infect Control* **4**. DOI

**Hawes JA, and Lee KS**. **2018**. Reduction in Central Line-Associated Bloodstream Infections in a NICU: Practical Lessons for Its Achievement and Sustainability. *Neonatal Netw* **37(2)**:105-115. DOI 10.1891/0730-0832.37.2.105.

**Huang FL, Chen PY, Chen YC, and Rehn YJF**. **2015**. Comparison of nosocomial infection rates in a neonatal intensive care unit before and after moving into a new location. *Journal of Microbiology, Immunology and Infection* **48(2)**:S178. DOI

**Huang H, Ran J, Yang J, Li P, and Zhuang G**. **2019**. Impact of MRSA Transmission and Infection in a Neonatal Intensive Care Unit in China: A Bundle Intervention Study during 2014-2017. *BioMed Research International* **2019**. DOI 10.1155/2019/5490413.

**Hussain AS, Ahmed AM, Arbab S, Ariff S, Ali R, Demas S, Zeb J, Rizvi A, Saleem A, and Farooqi J**. **2021**. CLABSI reduction using evidence based interventions and nurse empowerment: a quality improvement initiative from a tertiary care NICU in Pakistan. *Arch Dis Child* **106(4)**:394-400. DOI 10.1136/archdischild-2019-318779.

**Jain M, Meshram P, Bang A, Chauhan V, Datta V, and Dhanireddy R**. **2024**. Implementation of a quality improvement initiative for standardising essential newborn care in a teaching public hospital in rural central India. *BMJ Open Qual* **13**. DOI 10.1136/bmjoq-2022-001869.

**Johnson J, Latif A, Randive B, Kadam A, Rajput U, Kinikar A, Malshe N, Lalwani S, Parikh TB, Vaidya U, Malwade S, Agarkhedkar S, Curless MS, Coffin SE, Smith RM, Westercamp M, Colantuoni E, Robinson ML, Mave V, Gupta A, Manabe YC, and Milstone AM**. **2021**. Implementation of the Comprehensive Unit-Based Safety Program to Improve Infection Prevention and Control Practices in Four Neonatal Intensive Care Units in Pune, India. *Front Pediatr* **9**. DOI 10.3389/fped.2021.794637.

**Kallimath A, Patnaik SK, Malshe N, Suryawanshi P, Singh P, Gareghat R, Nimbre V, Ranbishe K, Kamble AG, and Ambekar V**. **2024**. Quality improvement initiative 'S-A-F-H' to reduce healthcare-associated neonatal sepsis in a tertiary neonatal care unit. *BMJ Open Qual* **13(Suppl 1)**. DOI 10.1136/bmjoq-2023-002336.

**Kawagoe JY, Dal Forno CB, Dornaus MF, Cunha LB, Santos MFC, Martins RAL, Portella MA, Climeni SMO, Petriccione S, Fernandes IM, Carvalho VS, Abreu MGB, Warth AN, Deutsch ADA, Correa L, and Abramovici S**. **2009**. Cultural and clinical changes in the NICU: Impact of a multi-faceted program on reduction of CVC associated BSI. *Am J Infect Control* **37(5)**:E121-E123. DOI 10.1016/j.ajic.2009.04.163.

**Kessler F, Coventry M, Albert C, and Douglass B**. **2011**. Preemie baseball game-implementing an innovative strategy of competition and team building to reduce bloodstream infections. *Am J Infect Control* **39(5)**:E209. DOI 10.1016/j.ajic.2011.04.041.

**Khurana S, Saini SS, Sundaram V, Dutta S, and Kumar P**. **2018**. Reducing Healthcare-associated Infections in Neonates by Standardizing and Improving Compliance to Aseptic Non-touch Techniques: A Quality Improvement Approach. *Indian Pediatr* **55(9)**:748-752. DOI 10.1007/s13312-018-1373-6.

**Koch AM, Nilsen RM, Dalheim A, Cox RJ, and Harthug S**. **2015**. Need for more targeted measures - Only less severe hospital-associated infections declined after introduction of an infection control program. *J Infect Public Health* **8(3)**:282-290. DOI 10.1016/j.jiph.2014.11.001.

**Ks D, Balachander B, and Rao Pn S**. **2021**. Feeding Practices, Lines, and Hospital-Acquired Infection during the Sustenance Phase of Infection Control Quality Improvement. *Journal of Pediatric Infectious Diseases* **16(1)**:26-30. DOI 10.1055/s-0040-1721445.

**Landre-Peigne C, Ka AS, Peigne V, Bougere J, Seye MN, and Imbert P**. **2011**. Efficacy of an infection control programme in reducing nosocomial bloodstream infections in a Senegalese neonatal unit. *J Hosp Infect* **79(2)**:161-165. DOI 10.1016/j.jhin.2011.04.007.

**Lee HC, Powers RJ, Bennett MV, Finer NN, Halamek LP, Nisbet C, Crockett M, Chance K, Blackney D, von Köhler C, Kurtin P, and Sharek PJ**. **2014a**. Implementation methods for delivery room management: a quality improvement comparison study. *Pediatrics* **134(5)**:e1378-1386. DOI 10.1542/peds.2014-0863.

**Lee SK, Shah PS, Singhal N, Aziz K, Synnes A, McMillan D, and Seshia MM**. **2014b**. Association of a quality improvement program with neonatal outcomes in extremely preterm infants: a prospective cohort study. *Cmaj* **186(13)**:E485-494. DOI 10.1503/cmaj.140399.

**Mazi WA, Bouafia NA, Kalarikkal TJ, Al Wagdani SH, and Abutaha RI**. **2019**. Controlling healthcare associated primary bloodstream infection in neonatal intensive care unit. *Antimicrob Resist Infect Control* **8**. DOI 10.1186/s13756-019-0567-6.

**McKechnie L, and Sethi K**. **2016**. A bundled approach to decreasing staphylococcal bacteremia in the neonatal unit. *Arch Dis Child* **101**:A247-A250. DOI 10.1136/archdischild-2016-310863.412.

**Mohamed Cassim S, Skiffington C, Lucas C, and Anand D**. **2015**. An improvement project to reduce central line associated blood stream infection (CLABSI) in newborn infants. *Arch Dis Child* **100**:A238-A239. DOI 10.1136/archdischild-2015-308599.491.

**Murray N, Pearson C, Loganathan P, Whyte M, and Cook J**. **2024**. REDUCING CENTRAL LINE-ASSOCIATED BLOOD STREAM INFECTIONS IN A TERTIARY NEONATAL UNIT: A QUALITY IMPROVEMENT PROJECT. *Arch Dis Child* **109**:A150-A151. DOI 10.1136/archdischild-2024-rcpch.219.

**Murthy V, Nath P, Millar M, Morris J, and Sinha AK**. **2014**. Prevention of late onset sepsis and central line associated blood stream infection in preterm infants. *Arch Dis Child* **99**:A56. DOI 10.1136/archdischild-2014-306237.134.

**Nankervis CA, Martin EM, Crane ML, Samson KS, Welty SE, and Nelin LD**. **2010**. Implementation of a multidisciplinary guideline-driven approach to the care of the extremely premature infant improved hospital outcomes. *Acta Paediatr* **99(2)**:188-193. DOI 10.1111/j.1651-2227.2009.01563.x.

**Neill S, Haithcock S, Smith PB, Goldberg R, Bidegain M, Tanaka D, Carriker C, and Ericson JE**. **2016**. Sustained Reduction in Bloodstream Infections in Infants at a Large Tertiary Care Neonatal Intensive Care Unit. *Adv Neonatal Care* **16(1)**:52-59. DOI 10.1097/ANC.0000000000000164.

**Nyantakyi E, Baenziger J, Caci L, Blum K, Wolfensberger A, Dramowski A, Albers B, Castro M, Schultes MT, and Clack L**. **2025**. Investigating the implementation of infection prevention and control practices in neonatal care across country income levels: a systematic review. *Antimicrob Resist Infect Control* **14(1)**. DOI 10.1186/s13756-025-01516-7.

**Okwujiako C, Scot M, Nath P, Bai C, Bullock K, Forde K, Guy C, North C, Sidgwick K, Toon J, and Williamson S**. **2024**. SEPSIS 'GOLDEN HOUR': QUALITY IMPROVEMENT INITIATIVE TO IMPROVE NEONATAL ANTIBIOTIC STEWARDSHIP PRACTICES. UNIVERSITY HOSPITAL OF COVENTRY AND WARWICKSHIRE. *Arch Dis Child* **109**:A135-A136. DOI 10.1136/archdischild-2024-rcpch.199.

**Oved M, Levi D, Ciobotaro P, Shinwell E, Bardestein R, and Zimhony O**. **2009**. Spiral of quality improvement: Prevention of nosocomial infections in a neonatal intensive care unit (NICU) in Israel. *Am J Infect Control* **37(5)**:E89. DOI 10.1016/j.ajic.2009.04.116.

**Pammi M, and Weisman LE**. **2015**. Late-onset sepsis in preterm infants: update on strategies for therapy and prevention. *Expert Rev Anti Infect Ther* **13(4)**:487-504. DOI 10.1586/14787210.2015.1008450.

**Patodia J, Mittal J, Sharma V, Verma M, Rathi M, Kumar N, Jain R, and Goyal A**. **2021**. Reducing admission hypothermia in newborns at a tertiary care NICU of northern India: A quality improvement study. *J Neonatal Perinatal Med* **14(2)**:277-286. DOI 10.3233/npm-190385.

**Perkins BS, Brandon DH, and Kahn DJ**. **2022**. Development of a Novel Assessment Tool and Code Sepsis Checklist for Neonatal Late-Onset Sepsis. *Adv Neonatal Care* **22(1)**:6-14. DOI 10.1097/ANC.0000000000000896.

**Pharande P, Lindrea K, Smyth J, Ward M, Lui K, and Bolisetty S**. **2014**. Trends in late onset sepsis in a neonatal intensive care unit following implementation of infection control bundle: A 10 year audit. *J Paediatr Child Health* **50**:102-103. DOI

**Polin RA, Denson S, Brady MT, Comm Fetus N, and Comm Infect D**. **2012**. Strategies for Prevention of Health Care-Associated Infections in the NICU. *Pediatrics* **129(4)**:E1085-E1093. DOI 10.1542/peds.2012-0145.

**Powers RJ, and Wirtschafter DW**. **2010**. Decreasing central line associated bloodstream infection in neonatal intensive care. *Clin Perinatol* **37(1)**:247-272. DOI 10.1016/j.clp.2010.01.014.

**Qadir M, Qamar FN, Resham S, Ali R, Khalil A, Ahmed S, Amin H, and Mossani S**. **2015**. Effectiveness of simple strategies in reducing multidrug resistant blood stream infections in Neonatal Intensive Care Unit of tertiary care hospital in Karachi, Pakistan. *Journal of the Pakistan Medical Association* **65(1)**:72-75. DOI

**Ramakrishnan K, Venkatesh S, Dhandapany G, and Palanisamy S**. **2015**. Infection control care bundles prevents emergence of multidrug resistant nosocomial pathogens in newborn care units: A perspective. *Journal of Global Infectious Diseases* **7(4)**:173-174. DOI 10.4103/0974-777X.170507.

**Ran NC, van den Hoogen A, and Hemels MAC**. **2019**. Gram-negative Late-onset Sepsis in Extremely Low Birth Weight Infants Is Emerging in The Netherlands Despite Quality Improvement Programs and Antibiotic Stewardship! *Pediatric Infectious Disease Journal* **38(9)**:952-957. DOI 10.1097/INF.0000000000002408.

**Rani U, Lewis L, and Chawla K**. **2020**. Development of an algorithm to prevent neonatal healthcare-associated infections (HAIs): A multimodal approach. *International Journal of Infectious Diseases* **101**:304. DOI 10.1016/j.ijid.2020.09.794.

**Ren ZX, Yang SM, Han JX, Nie CA, Wang CC, Wang JL, Zheng X, Yang HM, Zhang Q, Pei JJ, Xu F, and Yang J**. **2023**. Reduction of antibiotic use and multi-drug resistance bacteria infection in neonates after improvement of antibiotics use strategy in a level 4 neonatal intensive care unit in southern China. *EUROPEAN JOURNAL OF CLINICAL MICROBIOLOGY & INFECTIOUS DISEASES* **42(1)**:87-98. DOI 10.1007/s10096-022-04522-4.

**Resende DS, Jacqueline Moreira do O, de Brito DD, Abdallah VOS, and Gontijo Filho PP**. **2011**. Reduction of catheter-associated bloodstream infections through procedures in newborn babies admitted in a university hospital intensive care unit in Brazil. *Rev Soc Bras Med Trop* **44(6)**:731-734. DOI 10.1590/S0037-86822011000600015.

**Resende DS, Peppe ALG, dos Reis H, Abdallah VOS, Ribas RM, and Gontijo Filho PP**. **2015**. Late onset sepsis in newborn babies: Epidemiology and effect of a bundle to prevent central line associated bloodstream infections in the neonatal intensive care unit. *Brazilian Journal of Infectious Diseases* **19(1)**:52-57. DOI 10.1016/j.bjid.2014.09.006.

**Rogers E, Alderdice F, McCall E, Jenkins J, and Craig S**. **2010**. Reducing nosocomial infections in neonatal intensive care. *J Matern Fetal Neonatal Med* **23(9)**:1039-1046. DOI 10.3109/14767050903387029.

**Rohsiswatmo R, Rafika S, and Marsubrin PM**. **2014**. Prevention and control of blood stream infection using the balanced scorecard approach. *Acta Med Indones* **46(3)**:209-216. DOI

**Saiman L**. **2006**. Strategies for prevention of nosocomial sepsis in the neonatal intensive care unit. *Curr Opin Pediatr* **18(2)**:101-106. DOI 10.1097/01.mop.0000193300.25141.c5.

**Sanchez P**. **2010**. Strategies to limit infections in the neonate and to reduce infection-related mortality. *International Journal of Infectious Diseases* **14**:e181. DOI 10.1016/j.ijid.2010.02.1889.

**Savage T, Hodge DE, Pickard K, Myers P, Powell K, and Cayce JM**. **2018**. Sustained Reduction and Prevention of Neonatal and Pediatric Central Line-Associated Bloodstream Infection Following a Nurse-Driven Quality Improvement Initiative in a Pediatric Facility. *JAVA - Journal of the Association for Vascular Access* **23(1)**:30-41. DOI 10.1016/j.java.2017.11.002.

**Schulman J, Stricof RL, Stevens TP, Holzman IR, Shields EP, Angert RM, Wasserman-Hoff RS, Nafday SM, and Saiman L**. **2009**. Development of a statewide collaborative to decrease NICU central line-associated bloodstream infections. *J Perinatol* **29(9)**:591-599. DOI 10.1038/jp.2009.18.

**Scott C**. **2016**. A multi modal approach to reduce bloodstream infections in a large neonatal intensive-care unit. *Infection, Disease and Health* **21(3)**:116. DOI 10.1016/j.idh.2016.09.004.

**Sharma D, Murki S, Kulkarni D, Pawale D, Vardhelli V, Anne RP, Oleti TP, and Deshabhotla S**. **2020**. The impact of a quality improvement project to reduce admission hypothermia on mortality and morbidity in very low birth weight infants. *Eur J Pediatr* **179(12)**:1851-1858. DOI 10.1007/s00431-020-03711-7.

**Shepherd EG, Kelly TJ, Vinsel JA, Cunningham DJ, Keels E, Beauseau W, and McClead RE**. **2015**. Significant reduction of central-line associated bloodstream infections in a network of diverse neonatal nurseries. *Journal of Pediatrics* **167(1)**:41-46.e43. DOI 10.1016/j.jpeds.2015.03.046.

**Shettigar S, Somasekhara Aradhya A, Ramappa S, Reddy V, and Venkatagiri P**. **2021**. Reducing healthcare-associated infections by improving compliance to aseptic non-touch technique in intravenous line maintenance: A quality improvement approach. *BMJ Open Qual* **10**. DOI 10.1136/bmjoq-2021-001394.

**Short KL**. **2019**. Implementation of a Central Line Maintenance Bundle for Dislodgement and Infection Prevention in the NICU. *Advances in Neonatal Care* **19(2)**:145-150. DOI 10.1097/ANC.0000000000000566.

**Simeoni U**. **2011**. Quality improvement programs in neonatal care, including nosocomial infections. *J Perinat Med* **39**. DOI 10.1515/jpm-2012-1009.

**Singh HP, Wilkinson S, and Kamran S**. **2024**. Decreasing Antibiotic Use in a Community Neonatal Intensive Care Unit: A Quality Improvement Initiative. *Am J Perinatol* **41**:E2767-E2775. DOI 10.1055/a-2158-8422.

**Steiner M, Langgartner M, Cardona F, Waldhör T, Schwindt J, Haiden N, and Berger A**. **2015**. Significant Reduction of Catheter-associated Blood Stream Infections in Preterm Neonates After Implementation of a Care Bundle Focusing on Simulation Training of Central Line Insertion. *Pediatr Infect Dis J* **34(11)**:1193-1196. DOI 10.1097/inf.0000000000000841.

**Thor J, Herrlin B, Wittlöv K, Øvretveit J, and Brommels M**. **2010**. Evolution and outcomes of a quality improvement program. *Int J Health Care Qual Assur* **23(3)**:312-327. DOI 10.1108/09526861011029370.

**Wanigasekara R, Finch G, and Ponnusamy V**. **2023**. IMPROVING the RATES of LATE-ONSET PATHOGENIC BLOODSTREAM INFECTIONS in <32 WEEKS GESTATION PRETERM INFANTS THROUGH A QI APPROACH. *Arch Dis Child* **108**:A412-A413. DOI 10.1136/archdischild-2023-rcpch.645.

**Wheeler DS, Giaccone MJ, Hutchinson N, Haygood M, Bondurant P, Demmel K, Kotagal UR, Connelly B, Corcoran MS, Line K, Rich K, Schoettker PJ, and Brilli RJ**. **2011**. A hospital-wide quality-improvement collaborative to reduce catheter-associated bloodstream infections. *Pediatrics* **128(4)**:e995-e1004; quiz e1004-1007. DOI 10.1542/peds.2010-2601.

**Yasmeen A, Poole H, Jones B, and Lilley C**. **2011**. Trends in nosocomial infection rates over an 8 year period on a tertiary neonatal unit. *Archives of Disease in Childhood: Fetal and Neonatal Edition* **96**:Fa41. DOI 10.1136/archdischild.2011.300164.81.

**Zaka N, Alexander EC, Manikam L, Norman ICF, Akhbari M, Moxon S, Ram PK, Murphy G, English M, Niermeyer S, and Pearson L**. **2018**. Quality improvement initiatives for hospitalised small and sick newborns in low- and middle-income countries: a systematic review. *IMPLEMENTATION SCIENCE* **13**. DOI 10.1186/s13012-018-0712-2.

**Zini T, Miselli F, D'Esposito C, Fidanza L, Cuoghi Costantini R, Corso L, Mazzotti S, Rossi C, Spaggiari E, Rossi K, Lugli L, Bedetti L, and Berardi A**. **2024**. Sustaining the Continued Effectiveness of an Antimicrobial Stewardship Program in Preterm Infants. *Trop Med Infect Dis* **9(3)**. DOI 10.3390/tropicalmed9030059.
