# Supplementary material for: Quality improvement interventions to prevent late-onset sepsis in premature infants: a systematic review and meta-analysis
Source: PeerJ. 2026 Jan 2;14:e20530. doi: 10.7717/peerj.20530 (PMC12767489; doi:10.7717/peerj.20530)
Supplement: Supplemental Information 9 [file peerj-14-20530-s009.docx]

**The rationale for conducting the systematic review / meta-analysis**

Late-onset sepsis (LOS) is a life-threatening complication in preterm infants, with incidence rates ranging from 1% to 30% and varying due to clinical and geographical factors. It significantly increases the risks of mortality, acute brain injury, and long-term neurodevelopmental impairments in preterm neonates. Quality improvement (QI) methodologies hold the promise of bridging the gap between evidence-based best practices and real-world clinical outcomes, and QI bundles integrating multiple preventive measures show potential in reducing the risk of LOS. However, current systematic reviews either have a narrow focus on catheter-related bloodstream infections or include a mixed population of term and preterm infants, leaving a critical knowledge gap regarding QI strategies specifically tailored for preterm neonates. Moreover, the lack of quantitative synthesis across studies hinders the development of evidence-based guidelines. Thus, this systematic review and meta-analysis aims to synthesize the available evidence on the efficacy and safety of QI bundles in reducing LOS among premature infants, identify the key components associated with successful outcomes, and provide guidance for clinical practice and resource allocation to alleviate the global burden of this devastating complication.

**The contribution that it makes to knowledge in light of previously published related reports, including other meta-analyses and systematic reviews**

This meta-analysis is the first quantitative evaluation of the efficacy and safety of QI bundles in reducing LOS among preterm infants at birth, filling a crucial research gap. Through comprehensive synthesis and analysis, pooled odds ratios for different subgroups of preterm infants (VLBW and ELBW) are obtained, demonstrating that QI bundles can significantly reduce LOS. The study identifies core components such as multidisciplinary teamwork, staff education, hand hygiene optimization, and central line management that are consistently associated with the reduction of LOS across diverse settings, from high-income to resource-constrained environments. It also points out the substantial heterogeneity in the definitions of LOS and diagnostic criteria in previous studies, emphasizing the necessity of standardized reporting. Additionally, it analyzes the methodological limitations in previous studies, such as non-randomized before-and-after designs, and how these limitations can introduce biases. Furthermore, it reveals the impact of resource disparities on the implementation of QI bundles and the outcomes in different regions, as well as the structural inequities in neonatal care. Finally, it provides directions for future research, including conducting more studies in regions with a high burden of sepsis, adopting context-adaptive designs, integrating antibiotic stewardship into QI bundles, and standardizing the definition of LOS and the reporting of pathogen profiles.
